# A fully integrated harmonic injection and envelope-tracking architecture to extend the linearity of RF power amplifiers under high PAPR

**DOI:** 10.1038/s41598-025-28536-y

**Published:** 2025-12-05

**Authors:** Fazel Ziraksaz, Alireza Hassanzadeh

**Affiliations:** Faculty of Electrical Engineering, Shahid Beheshti University, Tehran, 1983969411 Iran

**Keywords:** Power amplifier (PA), Harmonic injection, 1-dB compression point, Hybrid modulator (HM), Envelope tracking (ET), High Peak-to-Average power ratio (PAPR), Energy science and technology, Engineering

## Abstract

High peak-to-average power ratio (PAPR) in modern modulation schemes causes power amplifier nonlinearity due to transistor saturation and results in considerable power loss. To mitigate these effects and extend the linear range, this work proposes a novel harmonic injection technique aimed at enhancing the 1-dB compression point. A new analytical model based on Taylor series expansion and drain current derivatives is developed, enabling a fully integrated injection scheme that avoids conventional components such as circulators or frequency doublers. A new biasing method is also derived from this model. To address power loss under high PAPR, a new fully CMOS-integrated hybrid envelope-tracking modulator is introduced. A custom-designed sensing circuit removes the need for sensing resistors and external voltage references, significantly reducing output ripple. Moreover, a new current management scheme is introduced that removes the ripple-filtering burden from the linear amplifier, simplifying its design. A comprehensive mathematical analysis is also provided to characterize the trade-offs between the inductor value and switching frequency, enabling a fully integrated on-chip inductor solution. Results in 180 nm CMOS show improvements of 1.2 dB in output saturation power, 4.4 dB in P_1dB_, and 12.5% in peak PAE. PAE at P_1dB_ improves by 14.6%, with EVM remaining below 5%.

## Introduction

The rapid evolution of modern wireless communication networks, driven by the exponential growth in mobile data traffic and the proliferation of connected devices, demands unprecedented performance in terms of speed, coverage, and energy efficiency^1–3^. These systems rely on complex modulation schemes with high peak-to-average power ratios (PAPRs) to meet the increasing demands for higher data rates and spectral efficiency^4,5^. However, high PAPR signals present critical challenges for radio-frequency (RF) power amplifiers (PAs). Specifically, they force the PA to operate under significant output power back-off to maintain linearity, reducing its efficiency^6^. Furthermore, the high peak amplitudes can push the PA into saturation, causing severe nonlinearity and limiting the linear output range—an issue that fundamentally impairs the overall transmitter performance^7^.

To address PA nonlinearity, various linearization techniques such as analog and digital predistortion, feedback, and feedforward have been developed^8–11^. Although these techniques improve linearity, they are accompanied by significant drawbacks, including higher system complexity, power dissipation in signal processing components, vulnerability to delay mismatches, and the requirement for extra hardware such as DACs and ADCs^10,11^. The Doherty PA, a well-known architecture designed to improve efficiency under power back-off conditions^11^, also presents implementation difficulties in CMOS technologies due to its dependence on distributed elements such as transmission lines, power combiners, and splitters. Furthermore, its narrow bandwidth limits its applicability in wideband systems^13^.

Given that the PA is the most power-hungry block in a transceiver, enhancing its efficiency is essential for power-conscious systems^14^. Nevertheless, a fundamental trade-off between linearity and efficiency exists in PA design, which is particularly critical in CMOS implementations due to constraints such as limited supply voltage, reduced power handling, and increased parameter variations at high frequencies^15^. Various approaches, including linear amplification with nonlinear components (LINC), aim to enhance efficiency, yet they frequently overlook the effects of high PAPR, resulting in degraded linearity or introducing integration complexities^16^. Thus, a strategy capable of jointly enhancing both linearity and efficiency is crucial for next-generation RF transmitters under high PAPR.

Variable power supply techniques, such as envelope tracking (ET) and envelope elimination and restoration (EER), have been introduced to address the power loss caused by operating PAs at a constant supply voltage under high PAPR conditions^17^. By dynamically adjusting the supply voltage according to the input signal envelope, these techniques reduce energy dissipation during back-off. ET has demonstrated better linearity compared to EER, but often suffers from limited efficiency due to the use of linear amplifiers in its main path^18^. To improve the trade-offs between bandwidth, efficiency, and integration, hybrid modulators have been proposed^19^. These architectures combine linear and switching amplifiers to simultaneously provide wide bandwidth and high efficiency, making them a promising candidate for variable supply PA systems^20^.

In recent years, harmonic injection has gained recognition as a promising approach for enhancing PA performance. The majority of existing harmonic injection architectures primarily aim at improving efficiency by minimizing the overlap between current and voltage waveforms or by suppressing third-order intermodulation distortion^21,22^. This technique aligns with the approach used in harmonic-terminated architectures like Class-F amplifiers, in which harmonic control at specific impedance nodes is employed to shape output waveforms and minimize the instantaneous overlap between voltage and current, thereby maximizing efficiency^23,24^. However, these systems typically require external components such as frequency doublers, phase shifters, diplexers, and circulators—elements that complicate the design, increase cost, and challenge integration in CMOS technologies. Moreover, sampling and amplifying the input signal to generate the second harmonic (SH) can reduce overall gain and introduce additional distortions. Despite the promising potential of harmonic injection techniques for linearity improvement, previous studies have largely overlooked a systematic investigation into their impact on linearity metrics—particularly the 1-dB compression point (1-dBCP), which is a critical indicator of amplifier linearity. This oversight underscores a substantial research gap that warrants dedicated exploration. Despite these limitations, harmonic injection remains a promising direction, particularly if its implementation can be fully integrated and refined for linearity enhancement.

In this work, a novel architecture is proposed that simultaneously improves linearity and efficiency of CMOS RF-PAs under high PAPR signals. To address the saturation-induced nonlinearity, a new second-harmonic injection method is developed based on an analytical model derived from Taylor series expansion of the drain current. This model guides the design of a fully integrated harmonic injection circuit that eliminates the need for external components such as frequency doublers and circulators. A new biasing strategy is also derived from the model to enhance the 1-dBCP, thereby expanding the PA’s linear operating region. To address efficiency degradation under high PAPR conditions, a new fully integrated hybrid envelope-tracking modulator (HETM) architecture is introduced. A custom-designed sensing circuit replaces conventional sensing resistors and external reference voltages, significantly reducing output ripple. Additionally, a novel current management scheme shifts the burden of ripple filtering away from the linear amplifier, simplifying its design. The proposed HETM also includes a comprehensive mathematical framework that models the relationship between the switching frequency and inductor value, allowing appropriate inductor selection for full on-chip integration.

The paper is organized as follows. Section 2 provides a concise review of conventional hybrid envelope tracking modulators (HETMs) and highlights their key limitations, particularly in handling high-PAPR signals. Section 3 introduces the proposed hybrid modulator architecture and presents a detailed theoretical analysis aimed at achieving high linearity and efficiency. Subsections 3.2 to 3.4 describe the design of the main building blocks: the linear amplifier designed for reliable operation under high-PAPR conditions, the sensing circuit section (including the sampler and current comparator), and the switching amplifier along with the relationship between inductor value and switching frequency. Section 4 presents the implementation and performance evaluation of the proposed power amplifier architecture and hybrid modulator. Contrary to conventional hybrid modulators, the proposed design enables the inductor to be implemented fully on-chip, supported by a thorough analysis of the relationship between inductor value and switching frequency, along with guidelines for selecting the optimum value. The hybrid modulator benefits from a novel current-sensing circuit that eliminates the need for sensing resistors, reduces power loss, and removes the requirement for external reference voltages. This compact structure allows supply tracking without relying on off-chip inductors or voltage references, making it fully integrable in CMOS technology. The power amplifier is based on an Internal Harmonic Injection (IHI) methodology, offering linearization with minimal complexity and high integration potential. Unlike conventional approaches, this design avoids DACs, ADCs, power combiners, frequency doublers, and circulators, enabling a fully CMOS-compatible, area-efficient implementation. Finally, Sect. 5 summarizes the main contributions and concludes the paper.

## Brief review of conventional hybrid envelope tracking modulators and their limitations

In ET systems, a dedicated modulator is required to dynamically supply the voltage for the main PA. These modulators are typically categorized into three types: linear, switching, and hybrid. The linear modulator offers wide bandwidth, which is advantageous for tracking rapid envelope variations of input signals. However, its primary drawback lies in its inherently low efficiency. For example^25^, demonstrates that although efficiency can be improved by approximately 5.5% using multiple parallel linear amplifiers, this approach significantly increases circuit complexity. On the other hand, switching modulators exhibit high efficiency but suffer from limited bandwidth, making them unsuitable for modern communication systems with high PAPR and wide signal bandwidths.

**Fig. 1:**
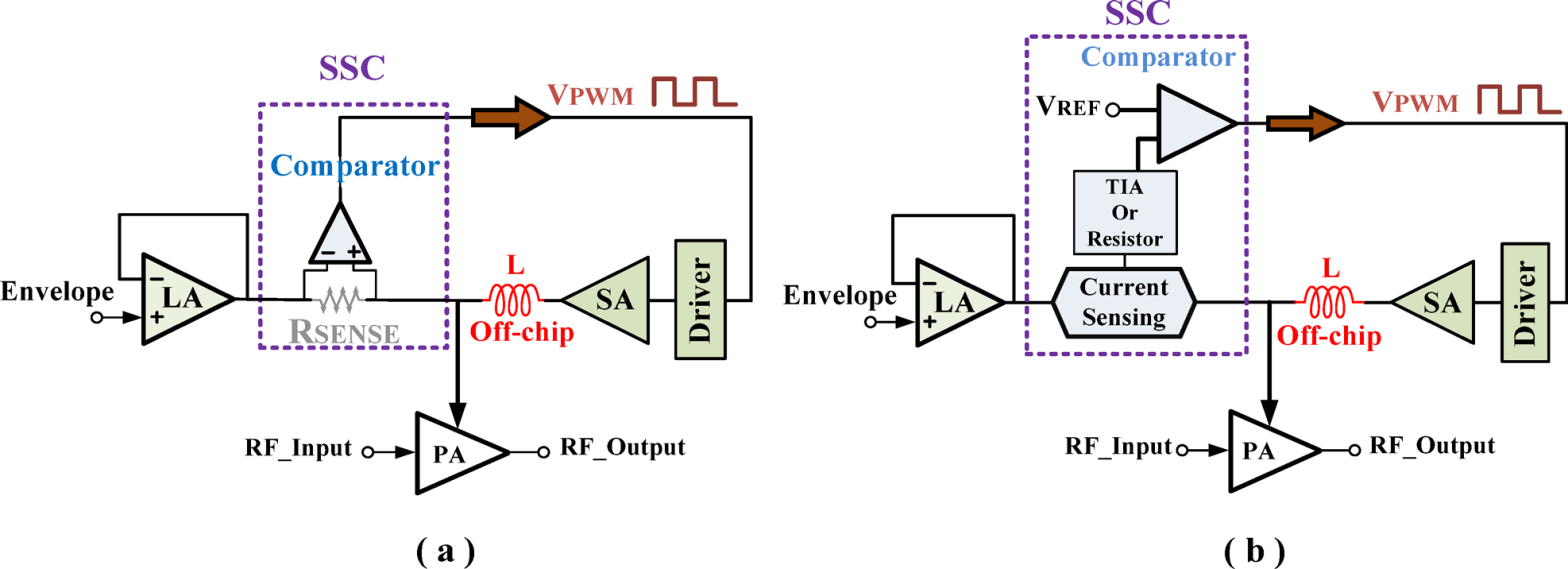
Conventional hybrid modulator architectures based on: (**a**) sensing resistor (R_SENSE_) (**b**) TIA or resistor.

To simultaneously achieve both high efficiency and wide bandwidth, hybrid modulators have been widely adopted. These modulators integrate a linear amplifier (LA) to accurately follow the input envelope and a switching amplifier (SA) to supply the majority of the current efficiently. The SA is typically controlled by a pulse-width modulated voltage signal (V_PWM_). Two conventional hybrid modulator topologies are commonly used to generate this V_PWM_, both of which are illustrated in Fig. 1. However, both suffer from increased structural complexity and elevated output ripple.

### Conventional topology using sensing resistor (R_SENSE_)

As depicted in Fig. 1(a), this structure incorporates a sensing resistor and a voltage comparator to form the sensing circuit. The output current from the LA flows through R_SENSE_, generating a voltage drop. This voltage is compared with a reference input using a comparator to produce the required V_PWM_ for the SA. The SA is responsible for delivering the majority of the output current. However, this topology inherently introduces considerable output voltage ripple and leads to increased power dissipation^26^. To address these issues, a two-phase modulation approach has been proposed. Nevertheless, this solution brings further complexity due to the addition of components such as ramp generators, current-sharing controllers, drivers, and inductors, which in turn limit the modulation bandwidth^27^.

### Conventional topology using current sensing via TIA or resistor

In the second approach shown in Fig. 1(b), the output current of the LA is converted into voltage using either a trans-impedance amplifier (TIA) or a resistor^28^. The resulting voltage is compared with a reference signal through a comparator to generate the V_PWM_ control signal for the SA. However, when a resistor is used as the current-to-voltage (I-to-V) converter, this structure faces similar challenges to the previous topology, such as increased ripple and reduced performance^29^.

Reference^30^ establishes the relationship between the switching frequency (f_SW_) and the value of R_SENSE_, as expressed by the following equation:

1 $$\:{f}_{SW\:}=\frac{{R}_{SENSE\:}\:\times\:\:{V}_{OUT}\:\times\:({V}_{DD}-{V}_{OUT})}{2\:{V}_{DD}NL\:{V}_{HYST}}$$

In Eq. (1), V_out_ represents the output voltage, V_DD_ is the supply voltage, N denotes the current mirroring ratio in the input stage, L is the inductor value, and VHYST corresponds to the hysteresis voltage. This relationship highlights how f_SW_ is directly influenced by R_SENSE_, contributing to the aforementioned limitations. Therefore, eliminating R_SENSE_ and redefining the switching frequency equation can help mitigate ripple and improve overall system performance.

In summary, conventional hybrid modulator topologies suffer from several limitations that hinder their full integration and performance. One major issue is the considerable ripple in the switching current, which is primarily filtered by the LA—thereby increasing its workload and reducing overall efficiency. Additionally, the sensing circuit itself introduces challenges, especially due to the voltage drop across the sensing resistor, which contributes to further ripple and accuracy degradation. Some designs attempt to overcome this by employing an external reference voltage; however, this approach compromises the feasibility of a fully on-chip implementation. Furthermore, these structures rely on off-chip inductors for energy storage, which not only increases system size but also eliminates the possibility of full integration.

## Proposed hybrid modulator architecture

To address the fundamental limitations of conventional hybrid envelope tracking modulators—including high output ripple, low integration, and excessive complexity—this work proposes a novel, fully integrated HM architecture that eliminates the need for external components, resistive sensing elements, and bulky compensation techniques.

**Fig. 2:**
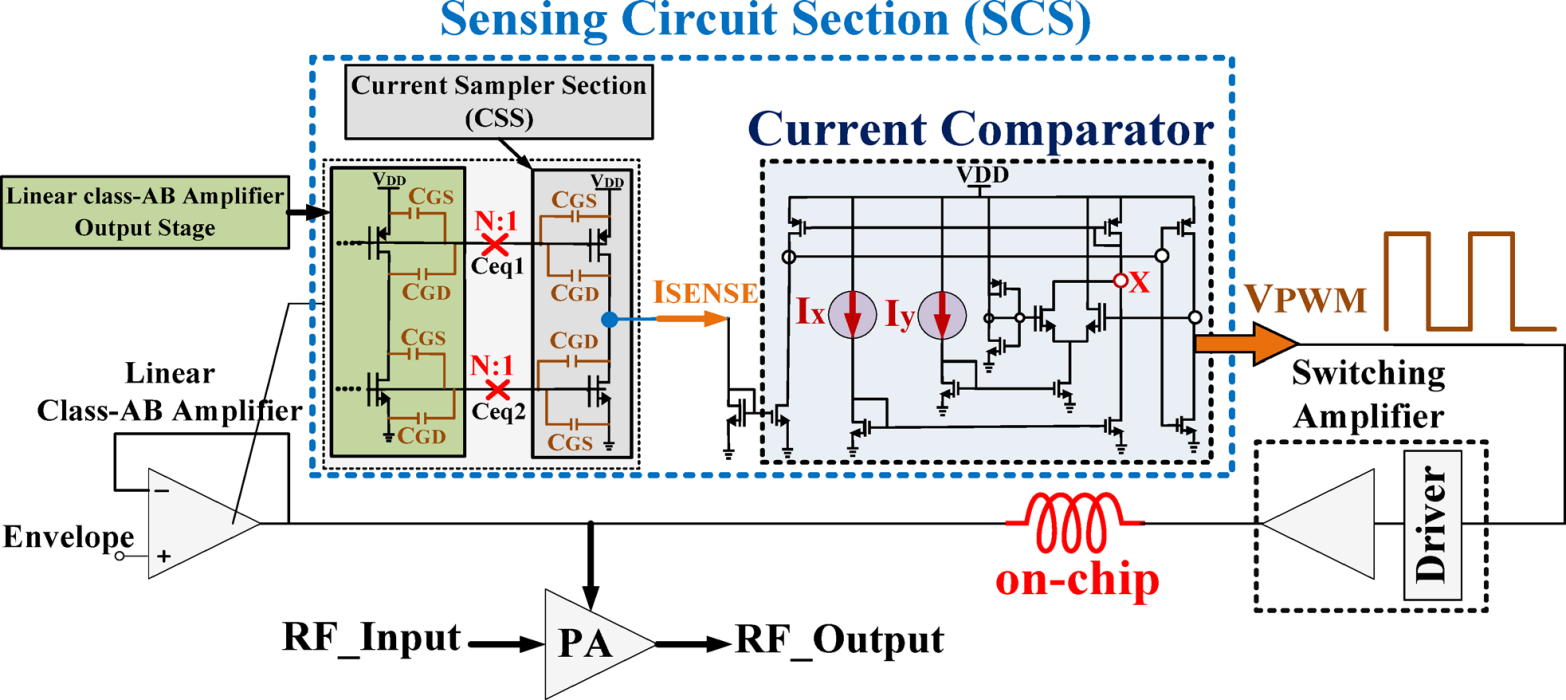
Proposed HM architecture.

The proposed architecture, illustrated in Fig. 2, consists of three core blocks: a wideband LA, an SA, and a simplified sensing circuit section (SCS). Unlike traditional HM implementations that rely on R_SENSE_, trans-impedance amplifiers (TIAs), external voltage references and off-chip inductors, this design achieves complete on-chip integration, significantly reducing power loss, area overhead, and system complexity.

To meet the demands of high-PAPR signals in modern RF systems, the wideband LA is designed with enhanced gain-bandwidth product and fast slew rate to enable accurate and linear envelope tracking. In typical designs, the sensing circuitry used to generate the V_PWM_ introduces voltage ripple and efficiency degradation due to the presence of lossy current-sensing resistors or voltage references. This work eliminates such elements by introducing a novel resistor-free current sensing scheme. Specifically, the output current of the LA is sampled by a current sampler section (CSS) and fed into a current comparator, which directly generates the V_PWM_ signal required to control the SA—without any intermediate voltage conversion or external reference sources. This not only improves tracking fidelity but also significantly reduces ripple and static power consumption.

Furthermore, to remove the dependency on off-chip inductors—which are common in previous HM structures for energy buffering and dynamic regulation—a dedicated analytical model is developed. This model enables the precise calculation of inductor values compatible with on-chip implementation, preserving fast dynamic response while enabling full integration in standard CMOS processes.

In summary, the proposed HM architecture introduces three key innovations:

1. A fully resistorless sensing mechanism, improving power efficiency and minimizing ripple.
2. An on-chip V_PWM_ generation technique without reference voltages or TIAs.
3. A compact, inductor-aware design approach that enables full CMOS integration.

These improvements support practical integration in modern wireless systems.

### Theoretical design considerations for high-performance hybrid modulators

#### Gain, bandwidth and stability

**Fig. 3:**
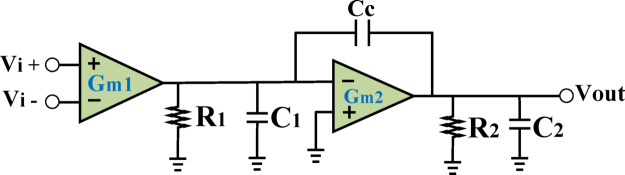
Two-stage amplifier structure.

To precisely track the envelope variations of the input signal, the LA—the first stage in the HM architecture—requires a high Gain-Bandwidth Product (GBW) and a high Slew Rate (SR)^31^. The modulator must achieve a bandwidth at least 2 to 6 times greater than the envelope bandwidth of the input signal to ensure accurate tracking without distortion^30,31^. Consequently, the LA must be designed to achieve a comparable bandwidth. Since a single-stage amplifier cannot typically meet the required gain, a two-stage structure is commonly adopted for the LA. In the two-stage LA configuration illustrated in Fig. 3, the overall gain is the product of the gains of each stage, expressed as A_stage1_ × A_stage2_ = G_m1_R_1_ × G_m2_R_2_ ​, where G_m1_ and G_m2_​ are the transconductance values of the first and second stages, respectively, and R_1_ and R_2_​ represent their output resistances.

The gain of the amplifier depends heavily on the output resistance of each stage, which is directly influenced by the channel length (*L*) of the transistors. Increasing *L* enhances the gain proportionally but decreases the amplifier’s bandwidth by a factor of ($\:\:\frac{1}{{L}^{2}}\:$), primarily due to increased parasitic capacitances. As a result, the GBW decreases with a factor of ($\:\:\frac{1}{L}\:$). Additionally, a higher *L* increases the overdrive voltage, reducing the output swing and potentially causing a loss of information. Therefore, selecting the minimum *L* that satisfies both gain and output swing requirements is critical to maximizing the BW and GBW, enabling the LA to precisely track the input envelope.

Phase Margin (PM) plays a pivotal role in determining the stability of multi-stage designs. In two-stage configurations, each stage introduces a pole. Typically, one pole is assigned as the dominant pole (P_d_), while the other becomes the non-dominant pole (P_nd_). The overall transfer function of the system is given by:

2 $$\:\frac{{V}_{out}\:}{{V}_{in}}=\:\frac{{(G}_{m1}{R}_{1}{)\:\times\:\:(G}_{m2}{R}_{2})\:}{\left(1\:+\:\frac{\mathrm{S}\:}{{P}_{d}}\:\right)\:\times\:\:\left(1\:+\:\frac{\mathrm{S}\:}{{P}_{nd}}\right)};\:{P}_{d}=\frac{1\:}{{R}_{1}{C}_{c}{G}_{m2}{R}_{2}};\:{P}_{nd}=\frac{{G}_{m2}\:}{{C}_{2}}$$

Where C_1_, C_2_ and Cc are the parasitic, load and compensation capacitance, respectively.

For the system to remain stable while maintaining satisfactory speed, the PM should fall within the range 45°< PM < 63.5°^32^. The placement of poles is therefore critical, as it directly affects both PM and GBW. For a two-stage structure, C_c_ and GBW are $\:\mathrm{tan}\phi\:\times\:\frac{{G}_{m1}\:}{{G}_{m2}}\times\:{C}_{2}$ and $\:\frac{{G}_{m1}\:}{{C}_{C}}$,respectively^32^. φ is the PM in degrees.

To enhance the GBW, the transconductance of the second stage (Gm2) is intentionally increased, which shifts the non-dominant pole. Implementing the compensation capacitor (CC) is challenging because on-chip CC consumes significant die area and off-chip solutions limit the full integration of the HM. This study overcomes these issues by introducing a novel technique that removes the requirement for CC, achieving adequate PM while minimizing die area and enabling a fully integrated, energy-efficient HM design.

#### Slew rate (SR)

A high slew rate (SR) is a critical parameter in the design of HMs as it directly impacts the ability to accurately track the signal envelope. An insufficient SR can severely hinder the tracking capability, resulting in nonlinearities that degrade system performance. According to^31^, the minimum required SR can be expressed as SR_min_= 2π × BW×0.5Vpp​, where BW represents the signal bandwidth and Vpp​ is the peak-to-peak voltage of the output signal.

In a two-stage amplifier architecture, the SR is predominantly determined by the first stage and can be approximated as $\:\frac{{\boldsymbol{I}}_{\boldsymbol{B}\boldsymbol{i}\boldsymbol{a}\boldsymbol{s}}}{{\boldsymbol{c}}_{\boldsymbol{c}}}$​​, where I_Bias_​ is the bias current of the first stage and C_C_ ​ is the compensation capacitor. Increasing I_Bias_​ enhances the SR but comes at the cost of increased power consumption, which can negatively affect the energy efficiency of the system. On the other hand, reducing C_C_ ​ improves the SR but may compromise system stability.

In HM designs, achieving an optimal SR is essential to balancing these trade-offs. A high SR ensures precise tracking of rapid envelope variations, particularly in signals with wide bandwidths, thereby preserving linearity and minimizing distortion. This work proposes a systematic approach to selecting I_Bias_ and C_C_ ​ values that achieve the desired SR while maintaining system stability and energy efficiency. By addressing these challenges, the proposed methodology paves the way for more reliable and efficient HM architectures tailored to modern wireless communication systems.

#### Output ripple

The output ripple is a critical parameter in HM architecture, as it directly impacts the system’s overall performance and signal fidelity. It is mathematically expressed as (I_SA_ × [Z_Load_ || Z_out _LA_]), where I_SA_ represents the switching current, Z_Load_ is the load impedance, and Z_out _LA_ denotes the output impedance of the LA. Minimizing the output ripple is essential to achieve a distortion-free signal and maintain the linearity of the HM. Among the contributing factors, the switching current typically has a substantial value, while the load impedance remains application-dependent and relatively constant. Consequently, the most practical and effective strategy for reducing output ripple lies in minimizing the output impedance of the LA. Designing the LA with adequately low output impedance allows the HM to effectively suppress output ripple, thereby maintaining signal integrity and enabling accurate envelope tracking. Such a design choice improves the linearity of the modulator while minimizing harmonic distortion, which is essential for high-performance applications.

### Proposed linear amplifier (LA) design for high-PAPR operation

**Fig. 4:**
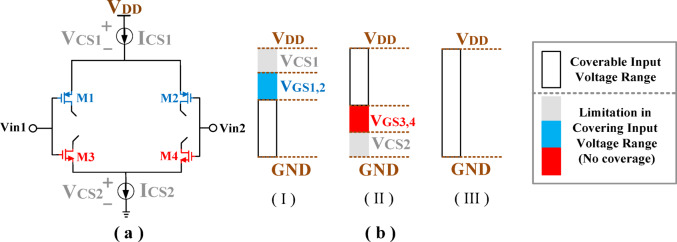
(**a**) Rail-to-rail amplifier (**b**) Coverable input voltage range: (I) PMOS (II) NMOS (III) Rail-to-rail pair.

For precise envelope tracking under high-PAPR conditions, the proposed LA requires a high gain-bandwidth product (GBW) along with a high slew rate (SR)^31^. In addition, to ensure linear operation and prevent distortion or information loss, the LA’s input stage must accommodate the entire input voltage swing ranging from 0 to V_DD_. To address this requirement, a rail-to-rail configuration is adopted that incorporates both NMOS and PMOS transistors in the input differential pair^51^, as illustrated in Fig. 4.a. Compared with conventional structures using only NMOS or PMOS devices (Fig. 4.b)^52^, this design provides a significantly wider linear input range. In contrast to prior studies that have largely neglected the importance of transconductance (G_m_) stability in HMs^25–31^, this work emphasizes the necessity of sustaining an almost constant G_m_ over the entire input range. This aspect is particularly vital for modern modulation formats with high PAPRs, where the envelope undergoes fast and wide variations. Through the integration of a rail-to-rail topology and Gm linearization, the proposed LA ensures reliable operation under challenging high-PAPR conditions, representing a central innovation of this design.

**Fig. 5:**
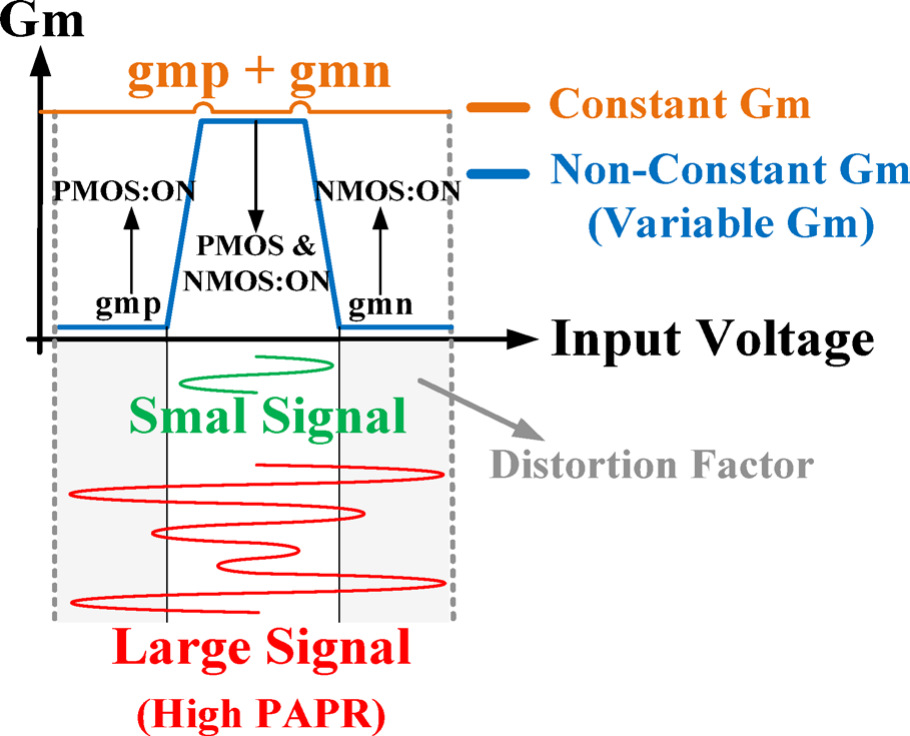
Total tranconductance of the rail-to-rail amplifier.

The major limitation in rail-to-rail architectures arises from distortion induced by variations in the total transconductance (g_m, tot_)^51^, as depicted in Fig. 5. This distortion becomes more significant under large input amplitudes and signals with high PAPRs^52^. Consequently, modern modulation schemes, which inherently exhibit high PAPRs, exacerbate this challenge. Although prior studies^43,53^ have attempted to widen the input range using rail-to-rail topologies, they largely neglected the critical requirement of maintaining a constant G_m_, which is essential for RF communication systems operating in high-PAPR environments^31^. In conventional rail-to-rail implementations, PMOS devices dominate at low input levels, whereas NMOS devices prevail at higher voltages. A crucial transition occurs in the intermediate region where both device pairs conduct simultaneously, thereby restricting the voltage range over which g_m, tot_ remains stable. Overcoming this drawback is fundamental to achieving high-linearity performance in RF front-ends. Several methods have been reported to stabilize G_m_^52^, typically through current injection or by maintaining a constant gate–source voltage when both NMOS and PMOS transistor pairs are active. However, despite their theoretical merit, these strategies have not been adopted in modulator design due to inherent limitations such as additional power consumption, increased input capacitance, and circuit complexity. In contrast, this work proposes a novel yet practical solution specifically tailored to modulator applications. The proposed approach ensures a nearly constant G_m_ within the LA stage while preserving architectural simplicity, thereby enabling improved linearity without incurring significant design overhead.

**Fig. 6:**
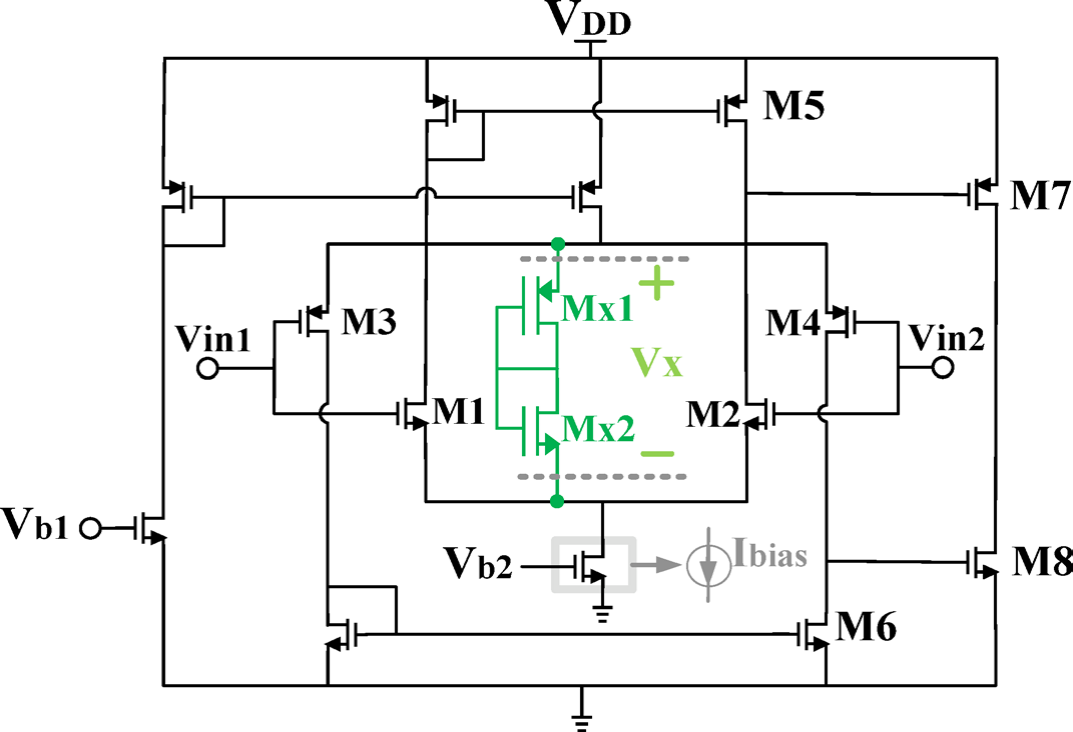
Proposed LA with constant g_m_.

To achieve constant G_m_, this work proposes a fundamentally different strategy, utilizing the structure shown in Fig. 6 as the first stage of the LA. The core innovation lies in the introduction of a floating voltage source (V_X_), which ensures the constancy of (V_GS, N_ + V_SG, P_), thereby stabilizing G_m, tot_​. The total transconductance is given by:

3 $$\:\mathrm{G}\mathrm{m},\mathrm{t}\mathrm{o}\mathrm{t}\:=\:\mathrm{G}\mathrm{m},\mathrm{N}\mathrm{M}\mathrm{O}\mathrm{S}\:+\:\mathrm{G}\mathrm{m},\mathrm{P}\mathrm{M}\mathrm{O}\mathrm{S}\:={\mu\:}_{n}{C}_{ox}{\left(\frac{W}{L}\right)}_{n}{(V}_{GS.N}-{V}_{th.N})+{\mu\:}_{P}{C}_{ox}{\left(\frac{W}{L}\right)}_{P}({V}_{SG.P}-\left|{V}_{th.P}\right|)$$

Where $\:{\mu\:}_{n}$, $\:{\mu\:}_{P}$, and $\:{C}_{ox}$ are the mobility of the NMOS, mobility of the PMOS and oxide capacitance, respectively. These parameters are process-dependent. To simplify this expression, it is assumed that ($\:{\mu\:}_{n}{C}_{ox}{\left(\frac{W}{L}\right)}_{n}={\mu\:}_{P}{C}_{ox}{\left(\frac{W}{L}\right)}_{P}=k$), which requires selecting appropriate $\:{\left(\frac{W}{L}\right)}_{n}$and $\:{\left(\frac{W}{L}\right)}_{P}$ ​values to satisfy this relationship. Consequently, the total transconductance simplifies to:

4 $$\:\mathrm{G}\mathrm{m},\mathrm{t}\mathrm{o}\mathrm{t}\:=k\:\left[{(V}_{GS.N}+{V}_{SG.P})-{(V}_{th.N}+\left|{V}_{th.P}\right|\:\right]$$

The value of V_X_ to maintain a constant G_m, tot_ is determined as:

To maintain a constant G_m, tot_​, the required floating voltage source is determined as:

5 $$\:\mathrm{V}\mathrm{X}\:=\frac{{G}_{m.tot}}{k}\:+{(V}_{th.N}+\left|{V}_{th.P}\right|)$$

To fully integrate HM architecture on a single chip, V_X_ is achieved by utilizing two diode-connected transistors, implemented by NMOS and PMOS transistors, as shown in Fig. 6. This particular approach can be applied to any other LA. The voltage across the diode-connected structure is determined as follows:

A key aspect of the proposed approach is its seamless integration into a single-chip HM architecture. The required V_X_​ is generated using two diode-connected transistors—one NMOS and one PMOS—forming the structure shown in Fig. 6. The voltage across this diode-connected pair is given by:

6 $$\:{V}_{X}\:=\:{V}_{SD.MX1}+\:{V}_{DS.MX2}\:=\:{(V}_{SG.MX1}-\left|{V}_{th.P}\right|)+\:{(V}_{GS.MX2}-{V}_{th.N})$$

The $\:\frac{W}{L}$ of each diode-connected transistor is determined as follows:

7 $$\:{\left(\frac{W}{L}\right)}_{MX1}=\:\frac{2\:{I}_{D}\:\:}{{\mu\:}_{p}{C}_{ox}{V}_{SD.MX1}^{2}}\:,\:{\left(\frac{W}{L}\right)}_{MX2}\:=\:\frac{2\:{I}_{D}\:\:}{\:{\mu\:}_{n}{C}_{ox}{V}_{DS.MX2}^{2}}$$

By determining the value of V_X_ and then the values ​​of V_DS_ and V_SD_ based on that, the size of the transistors is determined.

To meet the design objectives of high GBW product, high SR, and sufficient phase margin (PM), a two-stage rail-to-rail LA with a constant G_m_ ​ is proposed, as depicted in Fig. 6. Unlike prior methods, this approach offers a simple yet highly effective solution for achieving a stable transconductance, making it uniquely suitable for modern modulator architectures that operate under high-PAPR conditions.

**Fig. 7:**
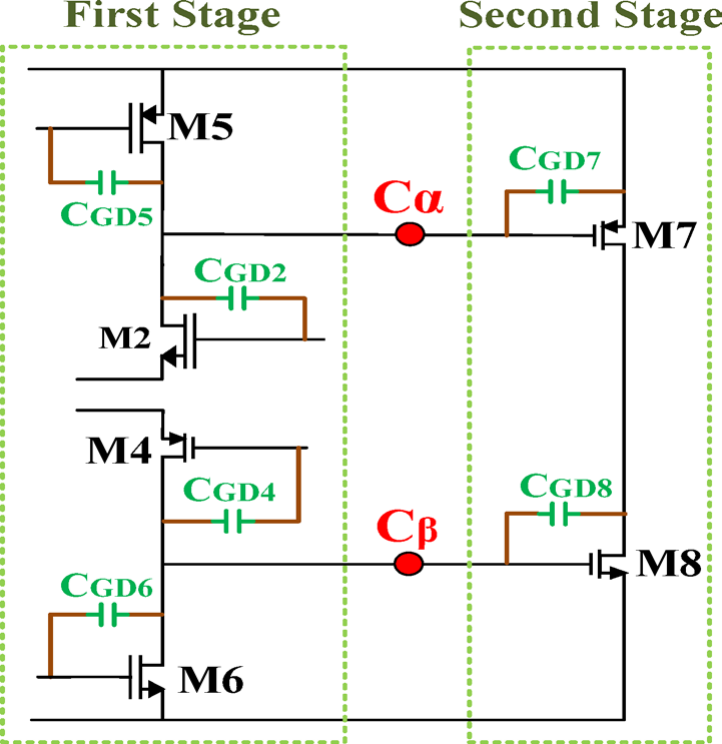
Equivalent capacitance between two stages.

To achieve high transconductance and output current at the LA output, large-size transistors are employed in the second stage. This approach also enhances the equivalent capacitance between the first and second stages due to the gate-drain capacitance, which is proportional to transistor width, as illustrated in Fig. 7 and Eq. (8). Notably, this increased capacitance acts as a built-in compensation capacitor, eliminating the need for external capacitors and enabling a fully integrated on-chip design.

8 $$C_{\alpha}=C_{GD7}+C_{GD5}+C_{GD2}; C_{\beta}=C_{GD8}+C_{GD4}+C_{GD6};\:{C}_{GD}\:\propto\:W$$

Conventionally, achieving the desired GBW and PM relies on the use of off-chip capacitors. The proposed design, however, eliminates the need for external components by employing an intrinsic capacitance compensation technique. By carefully adjusting the transistor widths, the equivalent capacitances are properly balanced, ensuring the target GBW and PM while preserving circuit simplicity. This innovative approach enables a compact and highly efficient implementation, effectively overcoming the limitations of conventional designs.

**Fig. 8:**
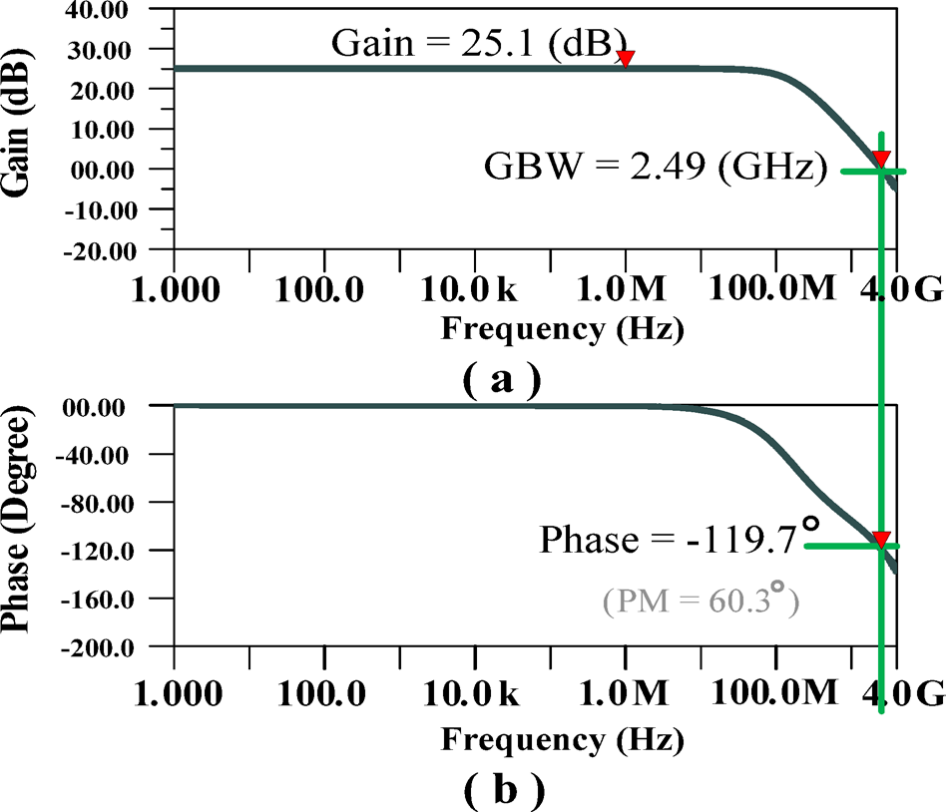
Frequency response of the proposed LA: (**a**) Gain and bandwidth of the structure (**b**) PM.

The efficacy of the proposed design is evident in its frequency response, as depicted in Fig. 8. The proposed architecture achieves a gain of 25.1dB and a bandwidth of 2490 MHz, significantly improving signal amplification across a broad frequency range. Additionally, as shown in Fig. 8b, the phase margin (PM) is 60.3°, ensuring stability and robust performance under varying operating conditions. The simulation results underscore the effectiveness of the proposed approach in achieving high gain and wide bandwidth without external compensation elements.

**Fig. 9:**
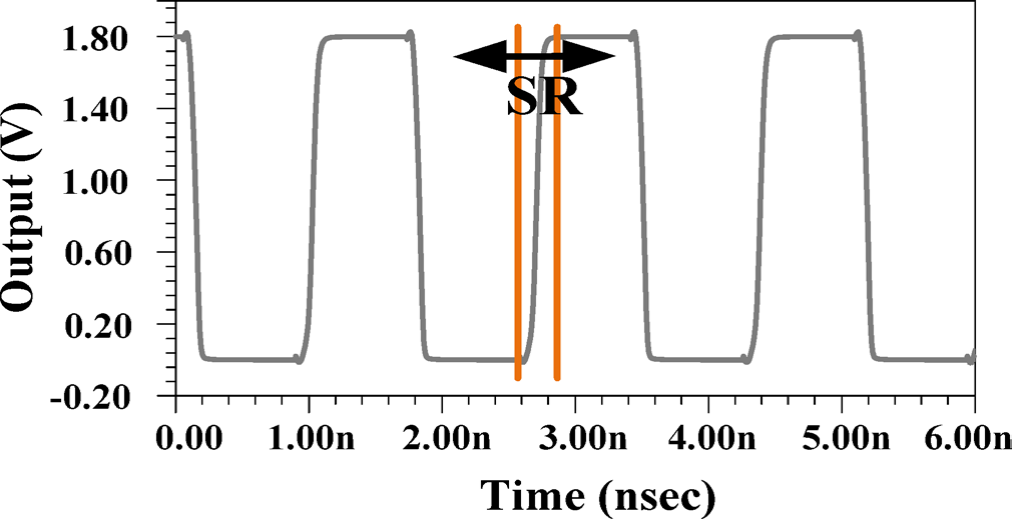
SR of the proposed structure.

A critical requirement for HMs is a sufficiently high SR to ensure accurate signal tracking. A low SR leads to signal distortion and non-linearity, especially in high PAPR applications. The theoretical minimum SR required for a 400 MHz bandwidth signal with a 1.8 V peak-to-peak output is 2.26 V/ns. However, the proposed structure far exceeds this requirement, achieving an SR of approximately 8.2 V/ns, as illustrated in Fig. 9. This remarkable enhancement ensures signal fidelity, making the design highly suitable for modern high-speed applications.

**Fig. 10:**
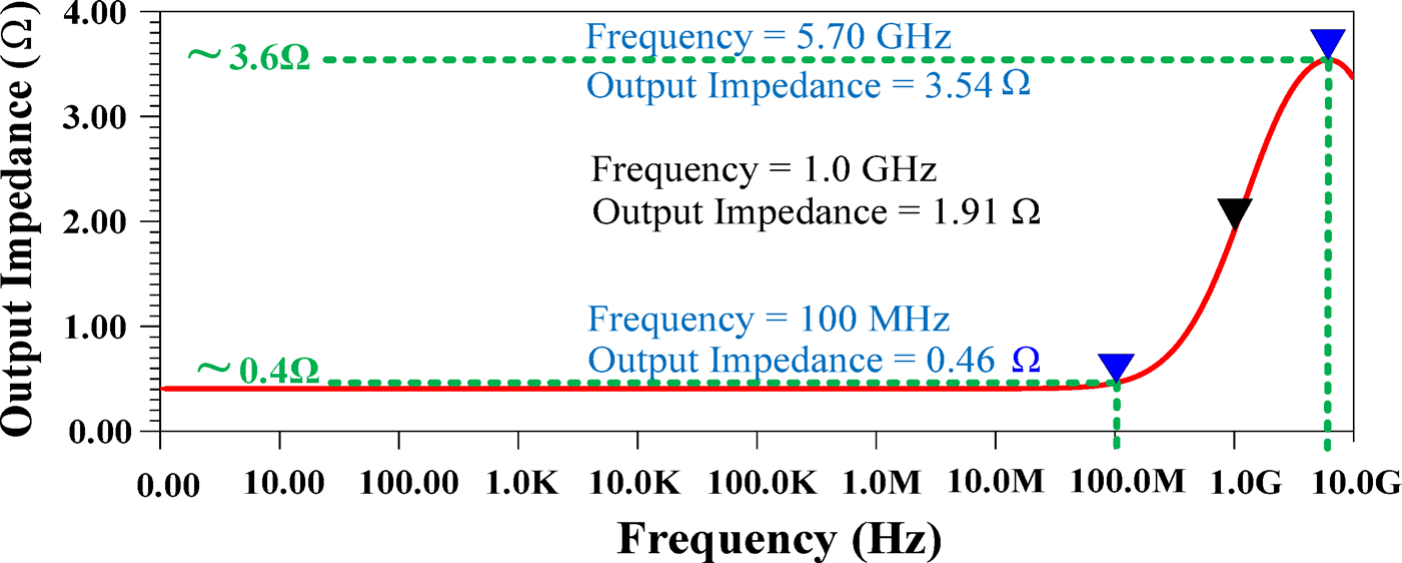
Output impedance of the proposed structure.

Maintaining a low output impedance is essential to minimize output ripple, especially in scenarios where switching currents are substantial, while load impedance remains application-dependent. The proposed design effectively achieves this by keeping the output impedance exceptionally low across a wide range of signal bandwidths. As shown in Fig. 10, the output impedance remains within 0.4Ω to 3.6Ω, ensuring stable operation and minimal ripple even under dynamic load conditions. This advantage is crucial for achieving high efficiency in envelope tracking and linear amplification applications.

**Fig. 11:**
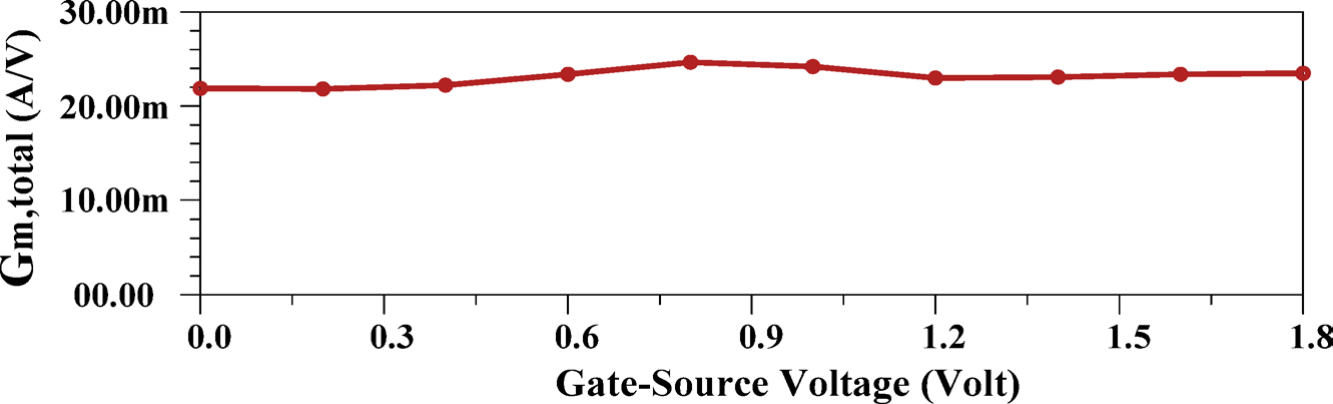
G_m_ of the suggested structure.

Figure 11 illustrates the G_m_ characteristic of the proposed structure. Unlike conventional designs, where G_m_ varies significantly, with the highest value exceeding twice the lowest value, the suggested architecture maintains an almost constant G_m_ with minimal fluctuations. This is a key innovation, as a stable G_m_ directly enhances linearity and efficiency, particularly in modulator applications that require precise amplitude control. The proposed approach, therefore, effectively mitigates non-linearity, ensuring optimal signal integrity and performance.

### Design of the proposed sensing circuit section (SCS)

**Fig. 12:**
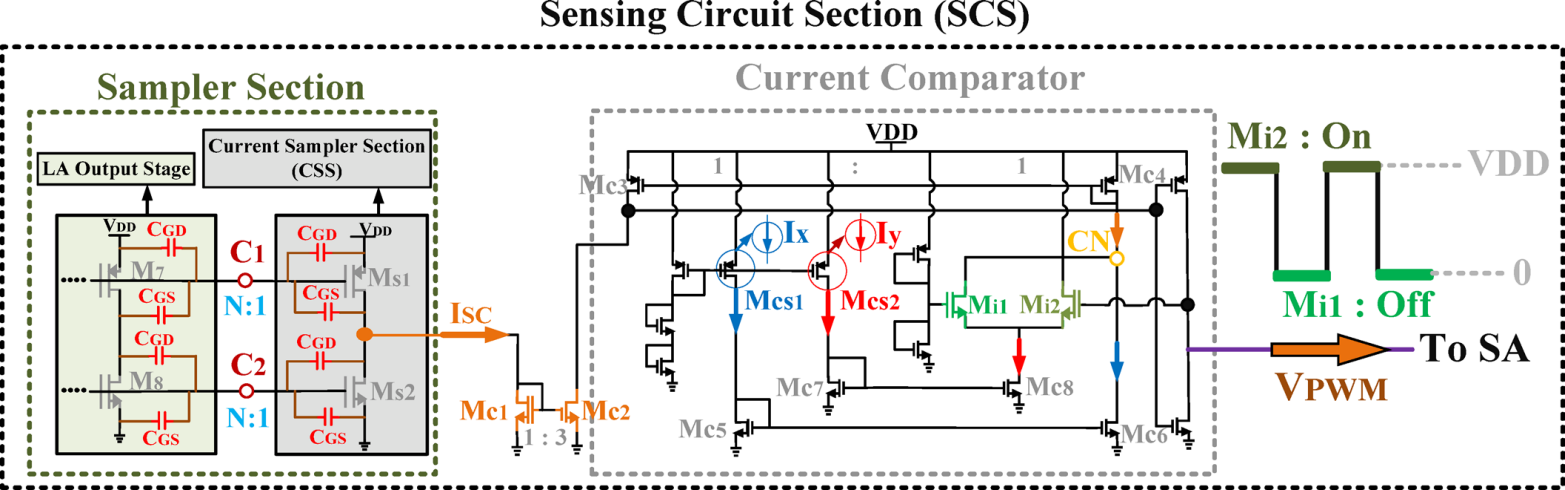
Proposed sensing circuit section (SCS) in the HM architecture.

The sensing circuit section (SCS) comprises the LA output stage, the current sampler section (CSS), and a current comparator, as illustrated in Fig. 12. This section plays a crucial role in the proposed HM architecture by interfacing between the LA and SA blocks and generating the V_PWM_ signal required for the SA circuit. The proposed current comparator is an advanced evolution of conventional comparators^35,36^, specifically enhanced to meet the requirements of the proposed HM. A key innovation of the proposed SCS lies in eliminating external components such as R_SENSE_ resistors, external reference voltages, and lossy resistor-based structures, which are commonly required in conventional architectures. Furthermore, the proposed design removes the necessity for current-to-voltage (I-to-V) conversion, as a current comparator is employed instead, addressing limitations in previous works. By integrating all essential components on-chip, this approach enhances efficiency, reduces complexity, and minimizes power losses, making it highly suitable for modern integrated circuits.

#### Sampler section

**Fig. 13:**
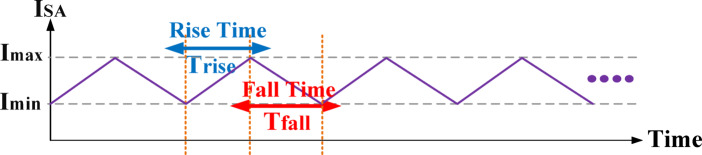
Currents waveform.

The sampler section comprises the LA output stage and CSS, which is responsible for sampling the LA output current at a predefined ratio, denoted by N, as shown in Fig. 13. A fundamental challenge in this section is the parasitic capacitance formed between these stages, which plays a crucial role in determining the sampling ratio. Additionally, this capacitance affects the system’s bandwidth and must be carefully addressed in the design. The relationship governing this capacitance is presented in Eq. (9).

9 $$\:{[{C}_{GD}+{C}_{GB}+{C}_{GS}]}_{M7}+{[{C}_{GD}+{C}_{GB}{+C}_{GS}]}_{Ms1};\:{[{C}_{GD}+{C}_{GB}+{C}_{GS}]}_{M8}+{[{C}_{GD}+{C}_{GB}+{C}_{GS}]}_{Ms2}$$

To overcome this challenge and avoid increasing the equivalent capacitances, a larger value for *N* must be chosen, which will result in the reduction of the sizes of M_S1_ and M_S2_, which are show in Eq. (10). Since capacitances the gate-drain (C_GD_) ​ **and** gate-source (C_GS_) are proportional to transistor width, decreasing **M**_**S1**_ **and M**_**S2**_ minimizes their parasitic capacitances. Therefore, this choice will result in a reduction of the sizes of M_S1_ and M_S2_, as shown in Eq. (10). Consequently, the C_GD_ and C_GS_ capacitances for M_S1_ and M_S2_ decrease. In this design, *N* is selected to be approximately 100.

10 $$\:{\left(\frac{W}{L}\right)}_{Ms1}=\frac{1}{N}{\left(\frac{W}{L}\right)}_{M7\:}\:,\:{\left(\frac{W}{L}\right)}_{Ms2}=\frac{1}{N}{\left(\frac{W}{L}\right)}_{M8}\:;\:{\:{C}_{GD}.\:{C}_{GB}\:and\:C}_{GS}\:\propto\:W$$

#### Current comparator

To provide the required V_PWM_ for driving the SA, the proposed SCS architecture employs a current comparator. Initially, the CSS samples the LA output current and generates a sampled current (I_SC_). This sampled current is then compared with two reference currents, Ix and Iy, to generate two distinct voltage levels (0 and V_DD_) at the SCS output for the SA section, as shown in Fig. 12.

To determine these levels, this work introduces a specific method for setting Ix and Iy. In the proposed SCS, Ix and Iy are set equal, with both values chosen to be less than half of I_SC_. Consequently, their sum remains lower than the maximum I_SC_ value. The sampled current I_SC_ is fed into the current comparator via the current mirror transistors MC1 and MC2, while the current source transistors MCS1 and MCS2 generate Ix and Iy, respectively.

**Fig. 14:**
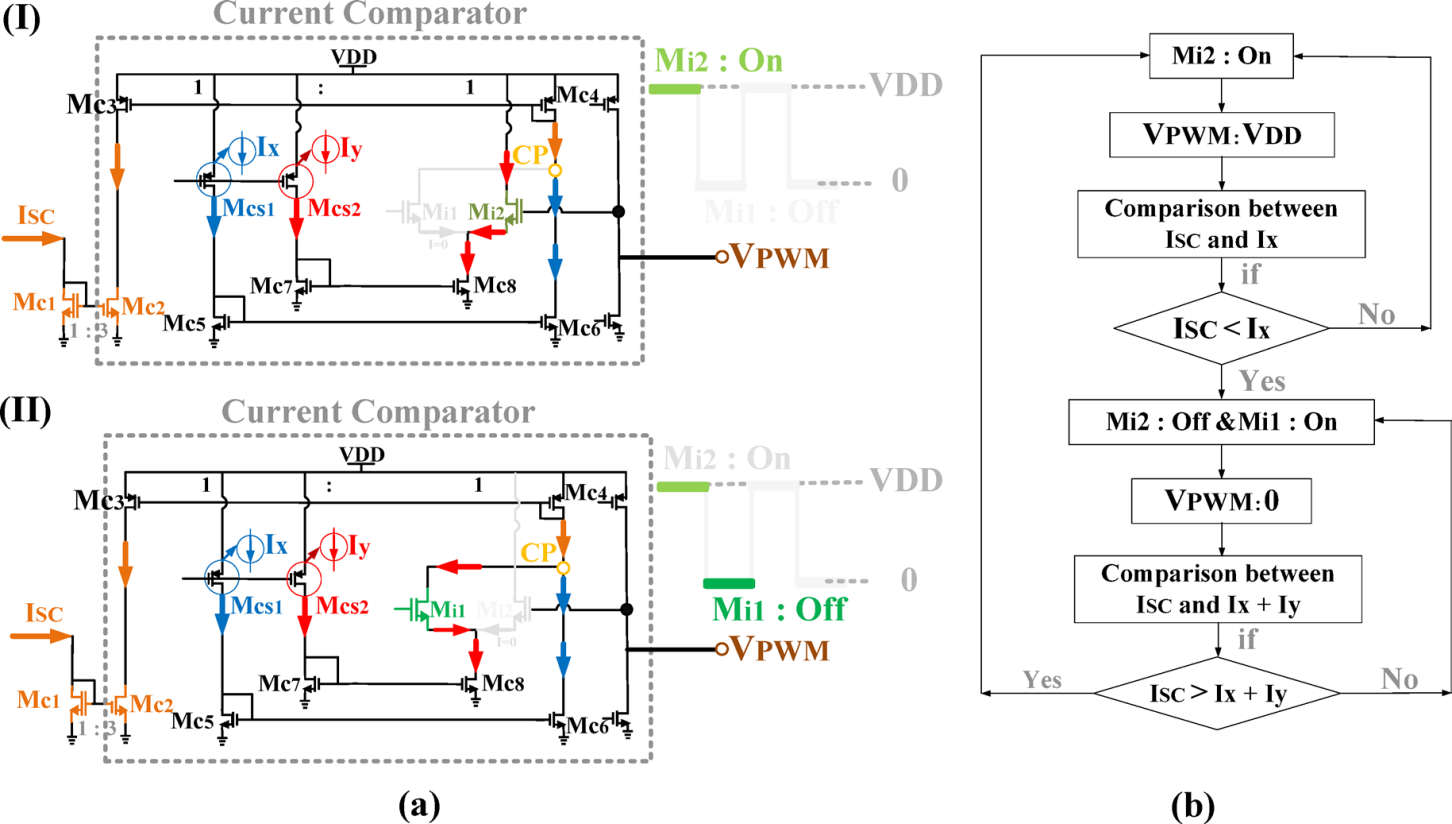
Proposed SCS architecture: (**a**) Operation of the simplified circuit (**b**) Flowchart of the suggested design methodology.

**Fig. 15:**
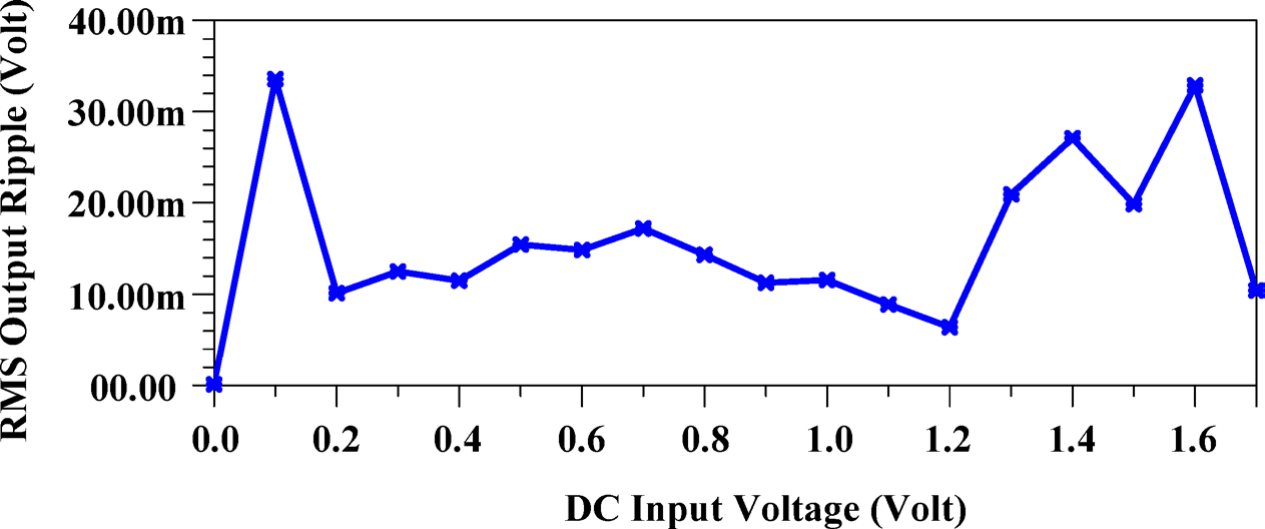
Output ripple of the suggested HM architecture.

The comparator performs the comparison in two steps. In the first step, I_SC_ is applied to the comparison node (CN) via the current mirror transistors. Transistor Mi2 is designed to be stronger than M_i1_, so M_i2_ turns on first, setting the comparator output to VDD. At this moment, M_i1_ sinks the entire I_y_ current, and the first comparison occurs between I_x_ and I_SC_ at node CN, as shown in Fig. 14.a.I. If I_SC_ is less than I_x_, M_i2_ turns off, lowering the V_PWM_ voltage level. In the second step, M_i1_ turns on, and the comparison shifts to I_x_+Iy versus I_SC_, as I_y_ enters node CN, as shown in Fig. 15.a.II. If I_SC_ exceeds I_x_ + I_y_, M_i2_ turns on again, restoring the high V_PWM_ level. This process continuously generates the required V_PWM_ signal with alternating voltage levels. The overall design methodology of the proposed SCS architecture is summarized in a flowchart, as illustrated in Fig. 14.b.

### Design of the switching amplifier and relation between the inductor value and switching frequency

**Fig. 16:**
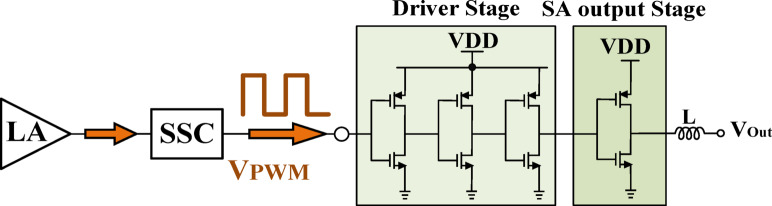
Switching amplifier structure.

Figure 16 illustrates the structure of the switching amplifier (SA) in the proposed HM architecture, which is responsible for delivering the primary portion of the output current.

One of the primary challenges in conventional HMs is ensuring that the LA effectively eliminates the switching ripple In the proposed design, this requirement is eliminated, significantly simplifying the overall design while improving precision. Since the LA is no longer required to remove switching ripple, the switching frequency is determined solely by the rise and fall times of the switching current. Unlike conventional designs, where the LA current significantly influences the switching frequency, in this work, the LA current is sinusoidal and of relatively high amplitude. Consequently, the SA assumes the primary role in defining the switching frequency, improving the accuracy of the mathematical model for frequency estimation. In traditional structures, aligning the switching ripple with the LA current to effectively cancel it presents a significant challenge. However, in the proposed structure, the SA maintains a nearly constant current with minimal variation between its peak and trough values. This allows the LA to supply a considerable sinusoidal current and contribute to the overall load current. Furthermore, in this design, the SR of the SA is intentionally set lower than that of the output current to improve overall performance.

A critical design trade-off exists between the inductor value (L) and the switching frequency (f_SW_). An increase in the inductor value leads to a decrease in the switching frequency. As expressed in Eq. (11), this reduction results in lower total power loss, primarily due to decreased switching losses:

11 $${P_{Loss}} = {\text{ }}{P_{SW}} + {\text{ }}{P_C};PSW = CeqfSW{V_D}{D^2};{P_C} = {R_{eq}}{I^2} = {\text{ }}{I^2}\left( {\left( D \right){\text{ }}{R_{on,{\text{ }}P}} + {\text{ }}\left( {1 - D} \right){\text{ }}{R_{on,N}}} \right)$$

Where P_Loss_, P_SW_ and P_C_ represent the total power loss, the switching power loss and the conduction power loss, respectively. C_eq_ and R_eq_ denote the total equivalent capacitance of the output stage of the SA and the total equivalent resistance of the output stage, including of the switch resistance of the PMOS (R_on, P_) and the switch resistance of the NMOS (R_on, N_). D, I and f_SW_ are the duty cycle, the average output current and the switching frequency.

By considering the output node currents of the LA, SA, and load, the SRs of these components are derived, providing key insights into their behavior. These relationships play a crucial role in determining the inductor value and switching frequency, directly impacting the overall performance of the system. Understanding these factors is essential for accurately modeling the proposed structure.

12 $$\:{{V}_{out}=A}_{m}{Sin(2\pi\:f}_{sw}\mathrm{t});\:{I}_{out}=\:{I}_{SA}+{I}_{LA}\rightarrow\:\frac{d{I}_{out}}{dt}=\:\:\frac{d{I}_{SA}\:}{dt}+\frac{d{I}_{LA}\:}{dt};\:{SR}_{out}={SR}_{LA}+\:{SR}_{SA}$$

13 $$\:\:\frac{d{I}_{out}\:}{dt}\:\:\frac{1}{{R}_{Load}}\frac{d{V}_{out}\:}{dt}\:{SR}_{SA}\:\frac{{V}_{PWM}-\:{V}_{out}}{L}\:{SR}_{LA}=\:\frac{1}{{R}_{Load}}\frac{d{V}_{out}\:}{dt}-\:\frac{{V}_{PWM}-\:{V}_{out}}{L}$$

Where L, V_out_ and R_Load_ are the inductor value, output voltage and load resistance, respectively.

In conventional HMs, large off-chip inductors are commonly used, which severely hinders full system integration and significantly reduces the SA’s output current slew rate. To counteract this, traditional approaches increase the LA’s output current. However, this solution comes at a major cost: due to the inherent efficiency limitations of the LA, higher output current leads to excessive power loss and reduced overall system efficiency^37^. This creates a fundamental trade-off, reducing the inductor size enhances integration but severely impacts SA performance, whereas increasing the LA’s current compensates for this at the expense of greater power consumption and efficiency degradation. The proposed work systematically tackles this challenge by introducing precise mathematical equations that define the necessary balance between switching frequency and minimum inductor value. This approach ensures both effective integration and improved performance, overcoming the limitations of conventional designs.

Figure 13 illustrates the current waveforms, highlighting the maximum and minimum current levels during the rising (T_rise​_) and falling (T_fall​_) intervals, which correspond to the SA switching on and off, respectively. The switching frequency is the inverse of the total cycle duration. The switching frequency can be determined using equations (14) to (17), as follows:

14 $$\:\mathrm{f}\mathrm{S}\mathrm{W}\:=\:\frac{1}{{\mathrm{T}}_{rise}+\:\:{T}_{fall}\:}$$

15 $$\:{\left(\frac{dI}{dt}\right)}_{Rise}\:=\:\frac{{I}_{max}-{I}_{min}}{{T}_{rise}}\:\:\:\:\:,\:{\:\:\left(\frac{dI}{dt}\right)}_{Fall}\:=\:\frac{{I}_{min}-{I}_{max}}{{T}_{fall}}$$

16 $$\:{SR}_{SA}=\frac{dI}{dt}=\frac{{{V}_{DD}\:-\:V}_{out}}{L}\:or\frac{{0\:-\:V}_{out}}{L}$$

17 $${T_{rise}} + {T_{fall}} = \:\:\frac{{I}_{max}\:-\:\:{I}_{min}}{\frac{0-{A}_{m}}{L}}\:\frac{\:{I}_{max}\:-{I}_{min}\:}{\frac{{{V}_{DD}-A}_{m}}{L}}$$

18 $$\:\mathrm{f}\mathrm{S}\mathrm{W}\:=\:\frac{1\:}{L}\:\frac{\:\left({{V}_{DD}-\:A}_{m}\right){A}_{m}}{\:\:({I}_{max}-{I}_{min})\:}$$

To accurately track the signal in the HM architecture, the output current slew rate must be faster than the maximum switching current slew rate. Furthermore, since the design goal is to implement an on-chip inductor that enables the complete integration of the proposed HM architecture onto a single chip, the inductor value must be less than 10nH. Given that the f_SW_ falls within the gigahertz range, it can be inferred that the impedance of both L and R_Load_ are likely to be in a similar range. Moreover, considering that V_out_ is a sinusoidal signal, represented as $\:{A}_{m}{Sin(2\pi\:f}_{sw}\mathrm{t})$, and V_PWM_ is a pulsed signal with values of 0 or V_DD_, $\:\left|{V}_{PWM}-{V}_{Out}\right|$ value will be between $\:{V}_{DD}-{A}_{m}$ and $\:{V}_{DD}+{A}_{m}$. Since SR_out_ is directly proportional to f_SW_, it can be inferred that SR_out_ will significantly exceed SR_SA_. Therefore, the assumption that SR_out_ is greater than SR_SA_ holds true. The minimum inductor can be determined using equations (19) to (21) along with the stated assumptions.

19 $$\:{SR}_{Out}\:=\:\frac{\partial\:{I}_{out}}{\partial\:t}=\frac{1}{{R}_{Load}}\:\frac{d{V}_{out}\:}{dt}\:=\:\frac{2{\uppi\:}{A}_{m}{f}_{SW}\:\mathrm{c}\mathrm{o}\mathrm{s}\left(2{\uppi\:}{f}_{sw}\mathrm{t}\right)}{{R}_{load}}$$

20 $$\:{SR}_{Out}=\frac{\partial\:{I}_{out}}{\partial\:t}=\frac{1}{{R}_{Load}}\:\frac{d{V}_{out}\:}{dt}\:>\:{SR}_{SA}=\frac{{V}_{PWM}-{V}_{out}}{\mathrm{L}}\:\:\:$$

21 $$\:{L}_{min}=\:\frac{{R}_{Load}\:\:{V}_{DD}}{2{\uppi\:}\:{f}_{sw}{\:A}_{m}\:}$$

Figure 15 presents the output ripple of the proposed HM, revealing that across an input voltage range of 0 V to 1.8 V, the average ripple remains approximately 15.3mV, with a minimum of 6mV at an input of 1.2 V.

**Fig. 17:**
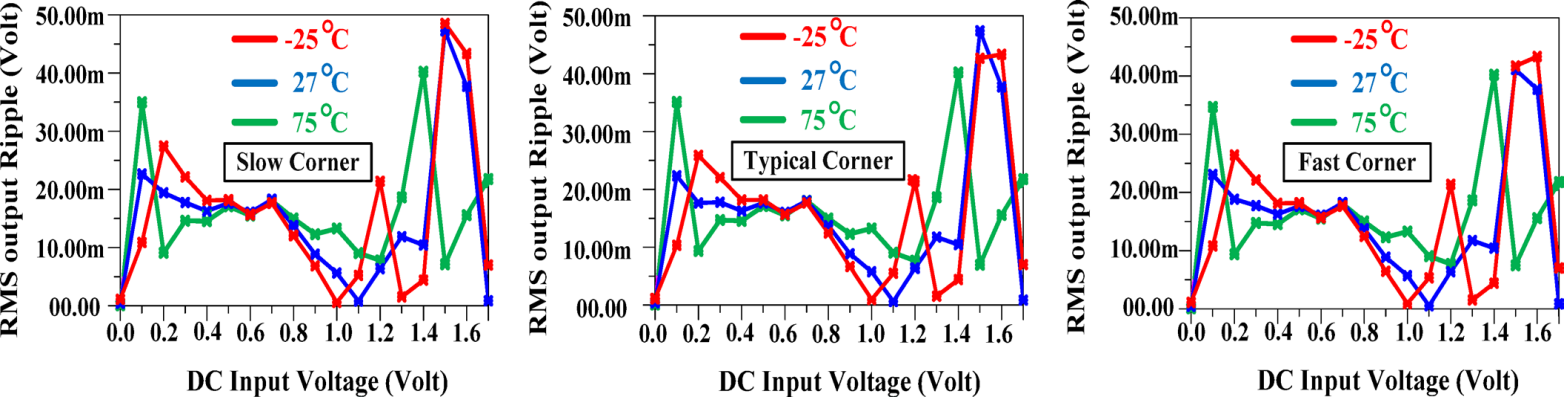
Output ripple in different corners and temperatures.

Figure 17 evaluates the architecture under different process corners and temperature variations. The simulation results demonstrate that the output ripple remains below 50mV across a wide input voltage range, even in extreme conditions. In the worst case, the average output ripple voltage is approximately 15mV, confirming the robustness of the proposed design.

**Fig. 18:**
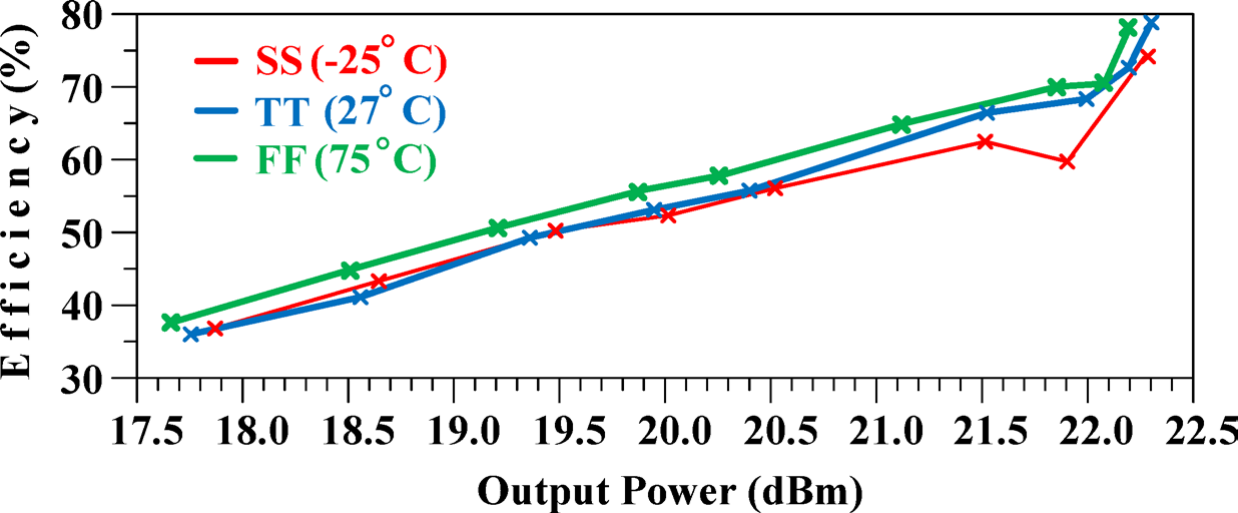
Efficiency of the suggested HM.

The efficiency of the proposed HM is shown in Fig. 18, with results indicating minimal variation across different conditions.

**Fig. 19:**
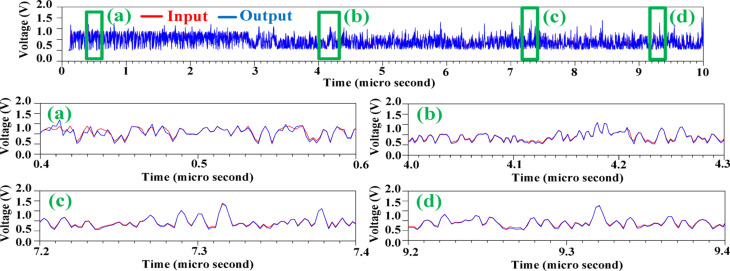
Tracked 64QAM signal with 11dB PAPR by the proposed HM.

Figure 19 demonstrates the performance of the proposed structure in tracking a 64-QAM signal with 11dB PAPR and a bandwidth of 200 MHz. The simulation results clearly show that the input signal is precisely followed, ensuring minimal distortion. This high level of accuracy in signal tracking directly contributes to a significant reduction in power loss, showcasing the efficiency of the proposed design even under demanding conditions. The simulation results validate the effectiveness of the architecture in delivering both high performance and energy efficiency, making it a candidate for practical applications in modern communication systems.

Table 1 presents a comprehensive comparison between the proposed architecture and existing HM structures, highlighting key differences and improvements. Conventional designs typically rely on resistor-based architectures, transimpedance amplifiers (TIAs), or digital control circuits incorporating ADCs and DACs. These approaches introduce significant complexity due to the need for multiple circuit components, such as ADCs, DACs, and additional compensation techniques to manage performance trade-offs. In contrast, the proposed architecture achieves a much simpler implementation while maintaining high performance.

A key advantage of the proposed HM is the elimination of off-chip components, particularly inductors, which are commonly used in conventional designs. Unlike prior works that rely on bulky off-chip inductors—limiting integration and increasing the overall footprint—this work employs an on-chip inductor, making it highly suitable for fully integrated solutions. The absence of off-chip elements not only enhances compactness but also improves power efficiency and scalability.

Moreover, the proposed design incorporates a constant gm structure, which plays a crucial role in achieving stable performance across different operating conditions. This feature ensures consistent transconductance, leading to improved linearity and more predictable behavior compared to conventional implementations, where gm variations can introduce nonlinearity and degrade performance.To provide a fair evaluation, the comparison in Table 1 considers implementations using the same technology node and similar application spaces. The proposed HM outperforms prior designs by achieving the highest figure of merit (FOM) and the widest bandwidth among the reported works, demonstrating its superiority in terms of both efficiency and integration.

**Table 1:** Comparison between the proposed HM and similar works.

Refs.	HM Architecture	Complexity	BW	Efficiency	Inductor Value	Output Power (dBm)	FOM ^16^
^27^	Sensing resistor	Moderate	20 MHz	83%	1 µH/0.3 µH	28.3	1.12
^29^	Current sense with TIA	Moderate	10 MHz	76.3%	----	29.66	0.71
^38^†	Current sensing and digital circuit	Very High	5 MHz	68.2%	0.15 µH	27.4	0.2
^39^†	Reference voltage	Simple	10 MHz	84%	0.75 µH	24	0.21
^40^	Sensing resistor and external reference voltage	Moderate	10 MHz	85%	----	28.9	0.66
^41^†	Digital circuit	Very High	5 MHz	73.5%	0.15 µH	26.8	0.18
^42^	Current sensing and sensing resistor	High	10 MHz	80%	1.1µH/2.2 µH	27	0.4
^43^†	Digital circuit	Very High	5 MHz	80.7%	0.5µH	32.17	0.67
This Work	Sensing circuit fully integrated	Moderate	200 MHz	79%	10nH	22.3	2.69

**†** Simulation results.

FOM = [bandwidth (Hz)]$\:\times\:$ [peak power (W)] $\:\times\:$ [peak efficiency ($\:\frac{\mathrm{p}\mathrm{e}\mathrm{a}\mathrm{k}\:\mathrm{e}\mathrm{f}\mathrm{f}\mathrm{i}\mathrm{c}\mathrm{i}\mathrm{e}\mathrm{n}\mathrm{c}\mathrm{y}\:\left(\mathrm{\%}\right)}{100}$)] $\:\times\:$ 10^− 7^^44^.

## Design of fully integrated harmonic injection p.a. with variable power supply voltage

In conventional harmonic injection studies, most efforts have focused on efficiency enhancement or third-order intermodulation (IM3) suppression. However, an equally critical challenge in PA design lies in the 1-dB compression point, where gain reduction leads to distortion and output saturation. For the conventional case without harmonic injection, this behavior can be modeled by expanding the drain current using a Taylor series and analyzing the contribution of higher-order terms. The linear gain is mainly determined by the first-order derivative, while gain compression originates from the third-order component. As established in the literature, particularly in Razavi^15^, the 1-dB compression point amplitude can be expressed as $\:{A}_{1dB.\:Conv}=\:\sqrt{0.145\left|\frac{{I}^{\left(1\right)}}{{I}^{\left(3\right)}}\right|}$. Here, only a brief overview of this conventional derivation is provided to give context, while the complete mathematical development is reserved for the proposed harmonic injection case, which constitutes the novel contribution of this work.

This section introduces a novel harmonic injection-based technique aimed at enhancing the performance of the PA, with particular emphasis on improving the 1-dB compression point, a key indicator of linearity. The proposed method employs a new analytical model derived using Taylor series to examine the internal harmonics and their influence on PA behavior. While prior studies have mainly concentrated on boosting efficiency by minimizing voltage–current overlap or mitigating IM3, the present work focuses on advancing linearity performance.

The PA is inherently nonlinear; therefore, its drain current I_D_ can be approximated using a Taylor series expansion as as $\:{I}_{D}\approx\:{I}_{0}+{I}^{\left(1\right)}{V}_{In}+{I}^{\left(2\right)}{V}_{In}^{2}+{I}^{\left(3\right)}{V}_{In}^{3}+\cdots\:+{I}^{\left(n\right)}{V}_{In}^{n}$, where I^(n)^ is the n-th derivative of the drain current with respect to the input voltage V_in_ (the gate-source voltage of the PA’s main core) and I_0_ represents the DC component. For a sinusoidal input $\:{V}_{in}={A}_{m1}Cos\left({\omega\:}_{1}t+{\phi\:}_{1}\right)$, the resulting amplitude of the main harmonic can be expressed as ($\:{I}^{\left(1\right)}{A}_{m1}+\frac{3}{4}{I}^{\left(3\right)}{A}_{1m}^{3}$), where the term ($\:\frac{3}{4}{I}^{\left(3\right)}{A}_{m1}^{3})$causes gain reduction. To address gain compression, it is essential to incorporate an additional term into the primary harmonic coefficient to prevent gain reduction and raise the 1-dB compression point (1-dBCP). This can be achieved by injecting the second harmonic into the input. The methodology presented here utilizes the internal second harmonic generated by the PA’s main core to enhance 1-dBCP, rather than removing harmonics through filtering. In this approach, the second harmonic produced at the PA output is fed back into the PA input along with the fundamental input signal via a dedicated injection path. Letting Am2 denote the amplitude of the second harmonic and ω2 its frequency, the input signal under injection conditions can be expressed as follows.

22 $$\:{V}_{in.inj}={A}_{m1}Cos\left({\omega\:}_{1}t+{\phi\:}_{1}\right)+{A}_{m2}Cos\left({\omega\:}_{2}t+{\phi\:}_{2}\right)\:\:;\:{\omega\:}_{1}=2{\pi\:f}_{1}\:\:\:;\:{\omega\:}_{2}=2{\omega\:}_{1}$$

In order to better observe the injection effect and simplify the estimations, the phase effect is ignored at this stage. The phase effect is important, and to overcome its challenges, this work employs time delay blocks. According to (22), the drain current after injection (I_D, inj_) is rewritten as follows:

23 $$\begin{aligned}\:{I}_{D.inj}={I}_{0}+({I}^{\left(1\right)}{A}_{m1}+{A}_{m1}{A}_{m2}{I}^{\left(2\right)}+\frac{5}{4}{I}^{\left(3\right)}{A}_{m1}^{3}+\frac{3}{2}{I}^{\left(3\right)}{{A}_{m1}A}_{m2}^{2}\:\\Cos(\omega_1{t})+(I^{(1)}Am_2+\frac{1}{2}I^{(2)}A^2m_1+\frac{3}{4}I^{(3)}A^3m_2+\frac{3}{2}I^{(3)}Am_2A^2m_1\\\:Cos(2\omega_1{t})+(\frac{1}{4}I^{(3)}A^3_{m1}+3I^{(3)}A_{m1}A^2_{m2}+A_{m1}A_{m2}I^{(2)})Cos(3\omega_1{t})\end{aligned}$$

In this method, with the second harmonic injection, the new coefficient of the main harmonic is given by ($\:{I}^{\left(1\right)}{A}_{m1}+{A}_{m1}{A}_{m2}{I}^{\left(2\right)}+\frac{5}{4}{I}^{\left(3\right)}{A}_{m1}^{3}+\frac{3}{2}{I}^{\left(3\right)}{{A}_{m1}A}_{m2}^{2}$). In the new coefficient, three terms are added to the linear gain ($\:{I}^{\left(1\right)}{A}_{m1}$) and the term ($\:\frac{3}{4}{I}^{\left(3\right)}{A}_{m1}^{2})$ is replaced, compared to the conventional structure. In fact, the compression point can be improved by tunning the parameters of these newly added terms. Considering that the *A*_*m2*_ coefficient is the amplitude of the second harmonic, to simplify (23), it can be considered as a coefficient of the main harmonic (*A*_*m1*_) and equals $\:\alpha\:$*A*_*m1*_, and (23) can be rewritten as follows:

24 $$\begin{aligned}\:{I}_{D.inj}={I}_{0}+\left({I}^{\left(1\right)}+\alpha\:{A}_{m1}{I}^{\left(2\right)}+\frac{5}{4}{I}^{\left(3\right)}{A}_{m1}^{2}+\frac{3{\alpha\:}^{2}}{2}{I}^{\left(3\right)}{A}_{m1}^{2}\right)\\{A}_{m1}\:Cos\left({\omega\:}_{1}t\right)+\:\left({\alpha\:I}^{\left(1\right)}+\frac{1}{2}{I}^{\left(2\right)}{A}_{m1}+\frac{3{\alpha\:}^{3}}{4}{I}^{\left(3\right)}{A}_{m1}^{2}+\frac{3\alpha\:}{2}{I}^{\left(3\right)}{A}_{m1}^{2}\:\right)\\{A}_{m1}\:Cos\left(2{\omega\:}_{1}t\right)+\:(\frac{1}{4}{I}^{\left(3\right)}{A}_{m1}^{2}+3{\alpha\:}^{2}{I}^{\left(3\right)}{A}_{m1}^{2}+\alpha\:{A}_{m1}{I}^{\left(2\right)}){A}_{m1}\:Cos\left({3\omega\:}_{1}t\right)\end{aligned}$$

**Fig. 20:**
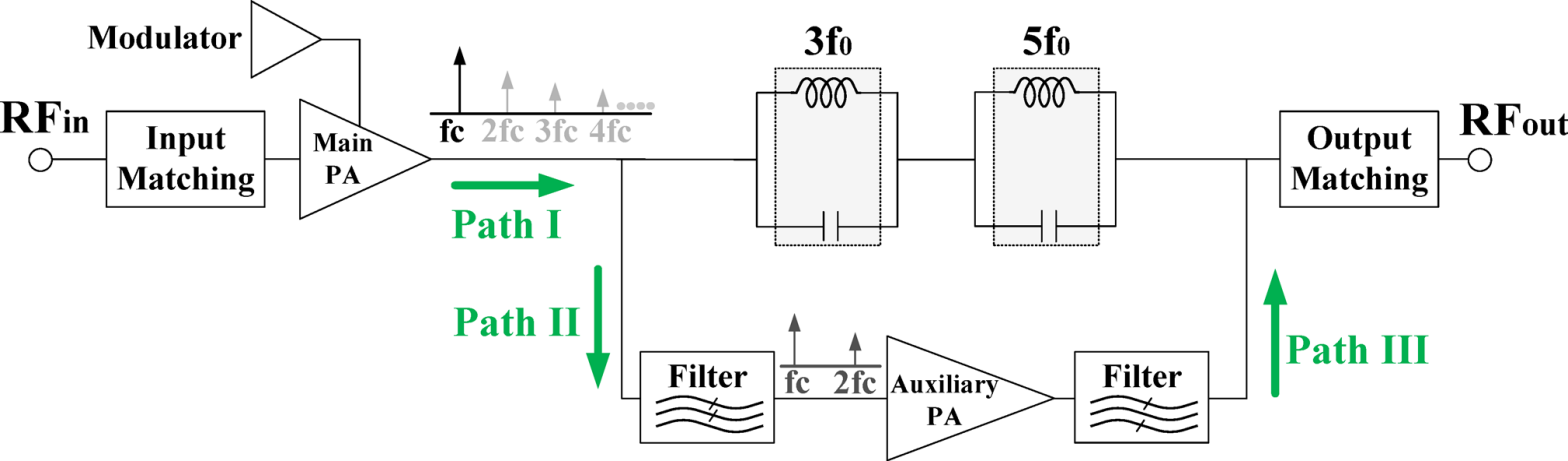
Block diagram of the proposed internal second harmonic injection methodology.

To implement the proposed methodology, a circuit structure is employed that applies the internal second harmonic (ISH) generated by the main core PA to the input of the harmonic injection PA’s main core. In other words, the 1-dBCP is improved by utilizing an auxiliary amplifier. The main and second harmonics of the main amplifier are applied as the inputs to the auxiliary amplifier, and the output of the auxiliary amplifier is fed into the main branch, thereby improving linearity where gain compression occurs. Figure 20 shows the block diagram implementation of the proposed methodology.

**Fig. 21:**
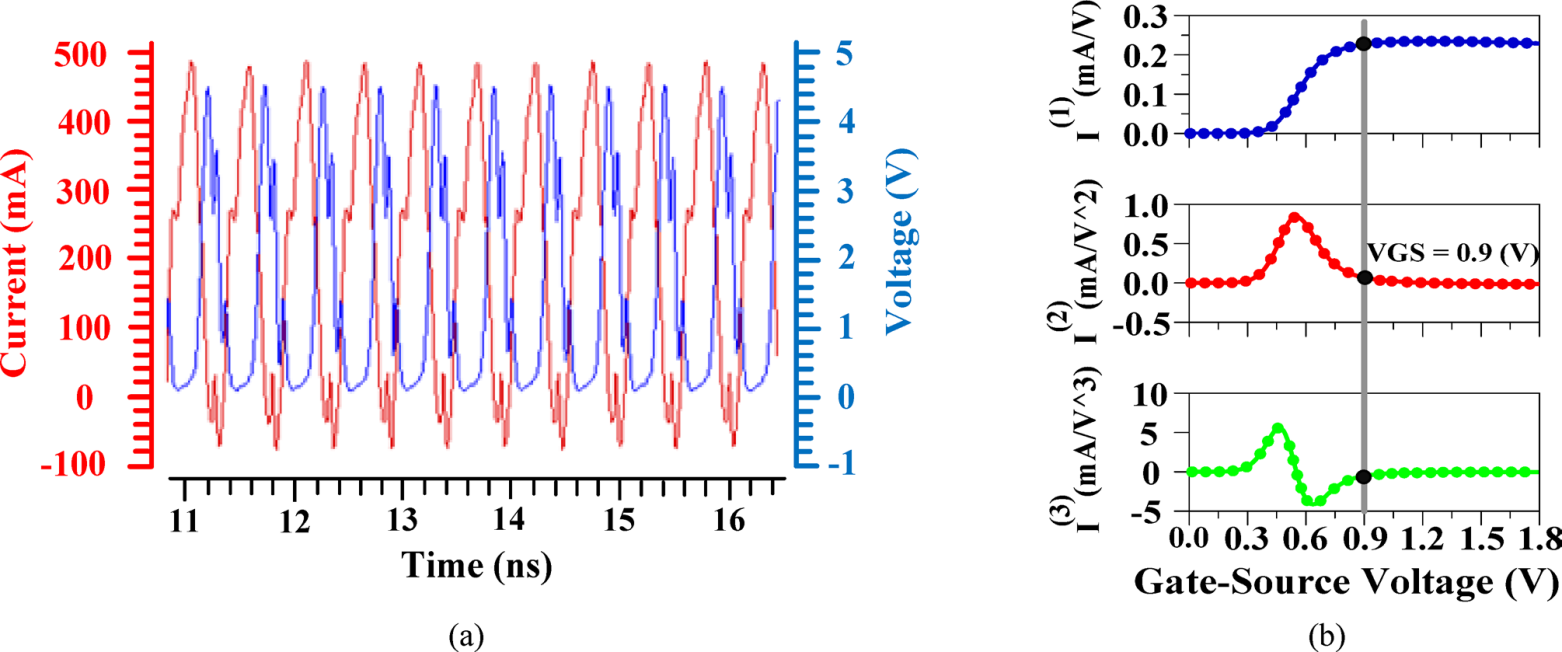
(**a**) Voltage and Current waveforms at the output; (**b**) $\:{I}^{\left(1\right)}$, $\:{I}^{\left(2\right)}$ and $\:{I}^{\left(3\right)}$ versus gate-source voltage curves.

Because the PA is a non-linear element, a set of harmonics is generated at the output terminal, with the first and second harmonics being stronger than the others, as shown in path I of Fig. 21. To select the desired harmonics, an LC filter is employed in path II, which allows only the selected harmonic frequency to pass through, blocking out-of-band frequencies. The third and higher-order harmonics are ignored due to their small amplitudes and are attenuated by the filter. In the filtering section of this work, LC tanks are used to selectively pass or block the harmonics. In path III, an LC tank tuned to the main harmonic frequency is used to prevent the main harmonic from entering the injection amplifier from path I. This approach eliminates the need for a circulator in the proposed structure, ensuring that all paths remain separated. Furthermore, since internal harmonics are used for injection, frequency doublers, which are commonly employed in conventional structures, are no longer necessary The removal of both the circulator and frequency doubler simplifies the PA circuit design and facilitates CMOS implementation. the sum of the currents from the main and auxiliary PAs is given by:

25 $$\begin{aligned}\:{I}_{DT}={I}_{D}+{I}_{D.inj}={I}_{0}+\left(2{I}^{\left(1\right)}+\alpha\:{A}_{m1}{I}^{\left(2\right)}+2{I}^{\left(3\right)}{A}_{m1}^{2}+\frac{3{\alpha\:}^{2}}{2}{I}^{\left(3\right)}{A}_{m1}^{2}\right)\\{A}_{m1}\:Cos\left({\omega\:}_{1}t\right)+\:\left({\alpha\:I}^{\left(1\right)}+{I}^{\left(2\right)}{A}_{m1}+\frac{3{\alpha\:}^{3}}{4}{I}^{\left(3\right)}{A}_{m1}^{2}+\frac{3\alpha\:}{2}{I}^{\left(3\right)}{A}_{m1}^{2}\:\right)\\{A}_{m1}\:Cos\left(2{\omega\:}_{1}t\right)+\:(\frac{1}{2}{I}^{\left(3\right)}{A}_{m1}^{2}+3{\alpha\:}^{2}{I}^{\left(3\right)}{A}_{m1}^{2}+\alpha\:{A}_{m1}{I}^{\left(2\right)}){A}_{m1}\:Cos\left({3\omega\:}_{1}t\right)\end{aligned}$$

Based on (25), 1dBCP for the injection mode is:

26 $$\:20\mathrm{log}\left(\left|2{I}^{\left(1\right)}+\alpha\:{A}_{m1}{I}^{\left(2\right)}+2{I}^{\left(3\right)}{A}_{m1}^{2}+\frac{3{\alpha\:}^{2}}{2}{I}^{\left(3\right)}{A}_{m1}^{2}\right|\right)\:=\:20\mathrm{log}\left(\left|{I}_{D}^{\left(1\right)}\right|\right)-1dB\:$$

27 $$\:{A}_{1dB.inj}=\frac{-\alpha\:{I}^{\left(2\right)}+\sqrt{{\alpha\:}^{2}{\left({I}^{\left(2\right)}\right)}^{2}-\left({I}^{\left(1\right)}{I}^{\left(3\right)}\right)(8\cdot\:8+6\cdot\:6{\alpha\:}^{2})}}{\left|\left(4+3{\alpha\:}^{2}\right){I}^{\left(3\right)}\right|}$$

**Fig. 22:**
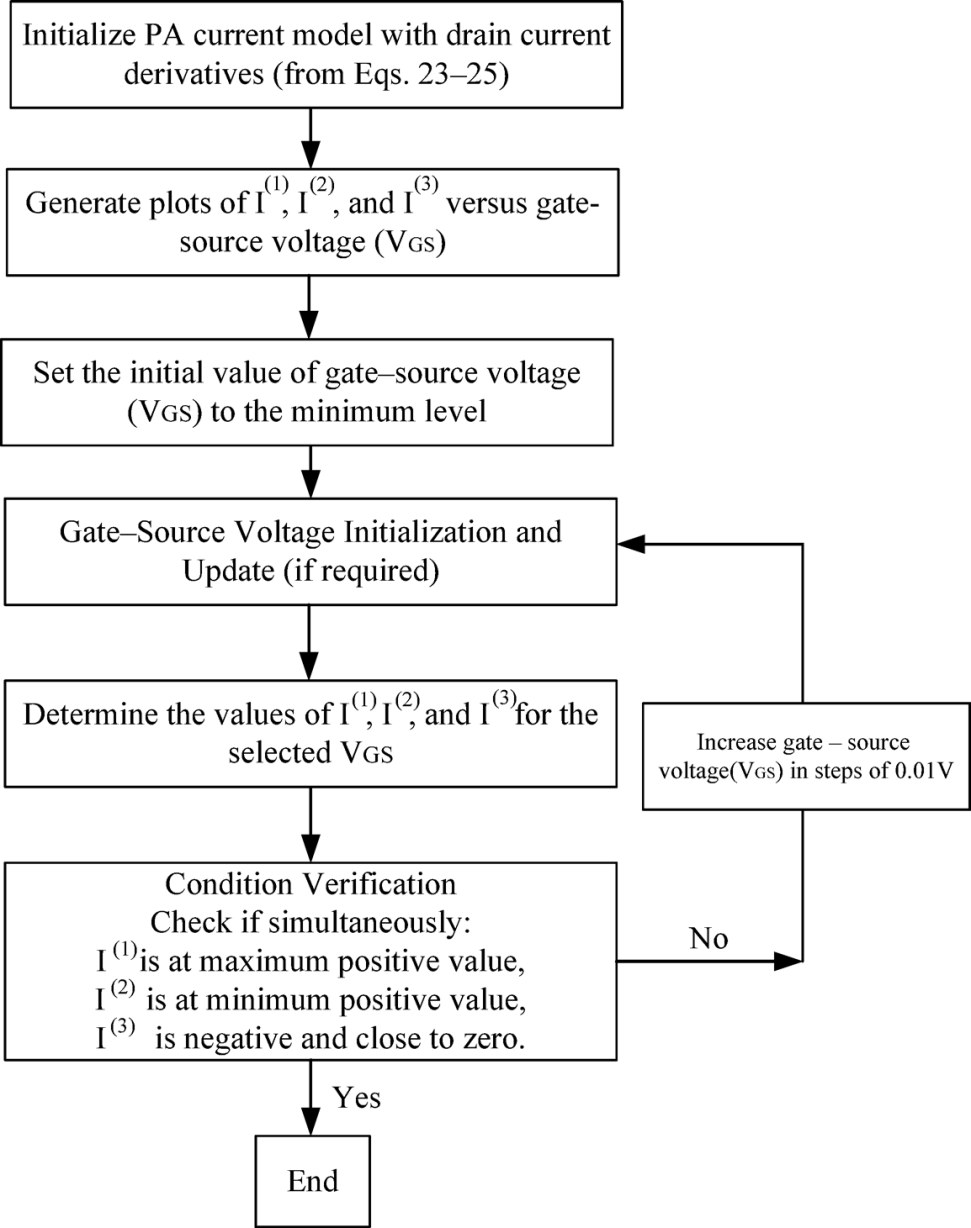
Flowchart for bias optimization in internal harmonic injection (IHI) architecture.

Equation (27) presents the key parameters for determining the *A*_*1dB, inj*_. Based on this equation, several options are available for biasing the PA. This work introduces a method to obtain the highest A_*1dB, inj*_ by adjusting these parameters. It should be noted that $\:\alpha\:$ represents the harmonic amplitude; however, the adjustment method introduced makes the value of the *A*_*1dB, injection*_ independent of $\:\alpha\:$. $\:{I}^{\left(1\right)}$, $\:{I}^{\left(2\right)}$, and $\:{I}^{\left(3\right)}$ represent the first, second, and third order derivatives of the drain current versus gate-source voltage, respectively. Based on Eqs. (25) and (27), and the main harmonic coefficient ($\:2{I}^{\left(1\right)}+\alpha\:{A}_{m1}{I}^{\left(2\right)}+2{I}^{\left(3\right)}{A}_{m1}^{2}+\frac{3{\alpha\:}^{2}}{2}{I}^{\left(3\right)}{A}_{m1}^{2}$), in order to achieve the highest 1-dBCP possible value, $\:{I}^{\left(1\right)}$, $\:{I}^{\left(2\right)}$ and $\:{I}^{\left(3\right)}$should be selected as a large positive value, a small positive value just above zero, and a negative value that is near zero, respectively. $\:{I}^{\left(2\right)}$ needs to be greater than zero to prevent gain compression. While $\:{I}^{\left(3\right)}$ needs to be just below zero to prevent damaging *A*_*1dB, inj*_. The selected value for $\:{I}^{\left(3\right)}$is determined by ensuring the radicand is positive, while also keeping the denominator of the fraction minimized to attain the highest possible 1-dBCP value. According to the specified conditions, the bias point of the PA is set to attain the maximum value of the 1-dBCP. These values ​​are obtained by adjusting the gate-source voltage value. The proposed methodology, Internal Harmonic Injection (IHI), represents an advancement in PA design by leveraging internal harmonics generated by the PA itself for harmonic injection. This innovative approach eliminates the necessity for external sources, streamlining the circuit design and enhancing efficiency. Furthermore, based on the mathematical relationships established in this work, a novel biasing technique for the PA is introduced, improving performance while maintaining simplicity. By focusing on internal harmonics, IHI not only improves linearity and reduces gain compression but also facilitates a more compact and integrated system architecture, marking a significant leap forward in the field of electronics. To provide a systematic bias optimization for the internal harmonic injection (IHI) technique, a flowchart is introduced in Fig. 22. The process begins by initializing the PA current model with drain current derivatives (from Eqs. 23–25). Next, the currents I^(1)^, I^(2)^, and I^(3)^ are plotted versus the gate–source voltage (V_GS_) to visualize their behavior. The minimum VGS is initially selected, and the plotted curves are inspected to check if all three conditions are simultaneously satisfied: I^(1)^ reaches its maximum positive value, I^(2)^ reaches its minimum positive value, and I^(3)^ reaches its minimum negative value. Achieving these conditions corresponds to the optimal bias that maximizes the 1-dB compression point according to Eq. 27. If the conditions are not met, V_GS_ is incrementally adjusted (e.g., in steps of 0.01 V), and the currents are re-evaluated until the optimal 1-dB compression point is reached. This methodology ensures a clear, reproducible, and systematic approach for bias optimization in the proposed IHI-based PA design.

**Fig. 23:**
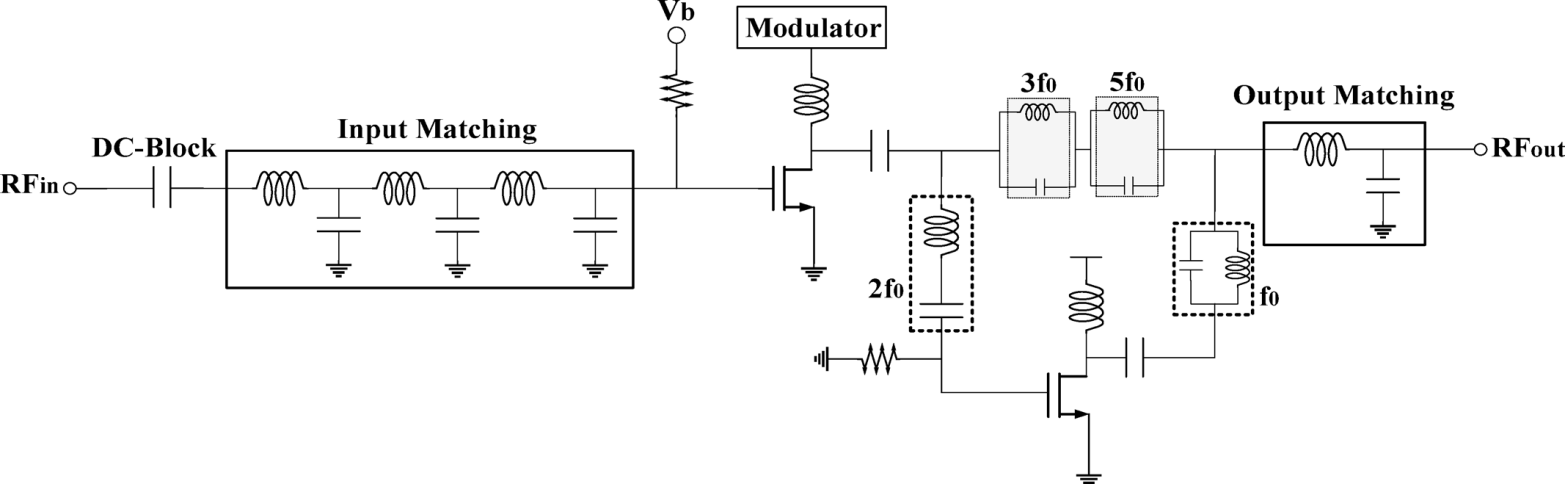
Fully integrated CMOS PA based on the proposed internal harmonic injection methodology.

Figure 23 shows the proposed fully integrated PA using the proposed methodology. The auxiliary PA section is responsible for injecting the internal second harmonic in the form of a current signal to the main amplifier at the point where gain compression begins. Due to the high impedance of the gate, the current at the input is combined with the main harmonic current. The resulting current is the sum of the main and second harmonics of the signal at the output. In the auxiliary circuit, the LC tanks block undesirable harmonic signals. The most important factors in the injection amplifier are the efficiency and power consumption. Therefore, the amplifier is biased in class C, meaning it will only be activated for the injection. Class C is ideal for architectures that require an additional amplifier to apply a signal effectively under specific conditions, such as in Doherty PAs. In the proposed approach, the injection section’s sole function is to inject the harmonic into the auxiliary PA, meaning it does not need to be on continuously like a Class-A PA^1,34]]^. It is only activated during the injection process. Additionally, to minimize the overlap between current and voltage waveforms, the third and fifth harmonic termination networks (HTNs) are employed in the proposed structure. Figure 21.a shows the current and voltage waveforms at the output, which shows that the overlap in the proposed circuit is limited. For biasing PA based on the proposed methodology, $\:{I}^{\left(1\right)}$, $\:{I}^{\left(2\right)}$, and $\:{I}^{\left(3\right)}$ should be set as shown in Fig. 21.b. According to the new method’s requirements, the gate biasing voltage for the main core is chosen to be 0.9 V.

**Fig. 24:**
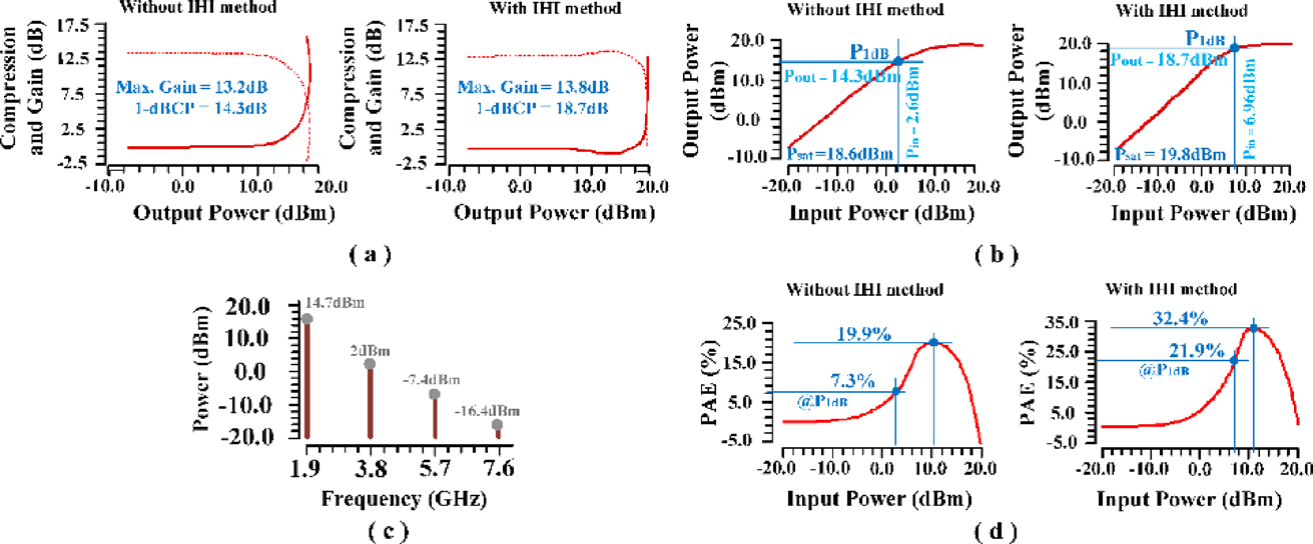
PA performance with and without proposed IHI method: (**a**) Power gain (**b**) output power and P_1dB_ (**c**) output power at different harmonics in main PA (**d**) PAE.

Figure 24.a presents the power gain and gain compression points achieved with and without the implementation of the proposed IHI technique. The results clearly indicate a significant enhancement in the 1-dB compression point, improving by approximately 4.4dB due to the application of the IHI method. Additionally, the overall gain has seen an increase of around 0.6 dB, underscoring the substantial efficacy of this innovative approach in enhancing PA performance. Figure 24.b further illustrates the output power and P_1dB_ for both scenarios, utilizing and not utilizing the IHI technique. The data reveals an impressive increase in output power by approximately 1.2dBm and a notable enhancement in P_1dB_ of about 4.4dBm. These findings underscore the transformative impact of the proposed IHI methodology on PA performance, highlighting its potential to significantly improve key metrics. In Fig. 24.c, the output power across different harmonics is examined. The results demonstrate that the power of the third and fourth harmonics is minimal, with further attenuation occurring through the filter, rendering them insignificant for practical considerations. In contrast, the main and second harmonics maintain considerable values. The filter is strategically configured to allow the second harmonic to pass while attenuating the first harmonic. Despite some attenuation, the first harmonic still retains a significant value, which is advantageous for the intended application of harmonic injection via the auxiliary amplifier. Figure 24.d illustrates the power added efficiency (PAE) before and after the implementation of the proposed IHI method. The findings reveal impressive improvements, with maximum PAE and PAE at P_1dB_ increasing by approximately 12.5% and 15%, respectively. This correlation between the rise in P_1dB_ and the enhancement in PAE further emphasizes the effectiveness of the IHI methodology in achieving higher efficiency while concurrently boosting amplifier performance. In summary, these results not only demonstrate the remarkable improvements realized through the proposed IHI method, but also underscore its effectiveness in enhancing power efficiency, increasing the 1-dB compression point, and facilitating a more compact amplifier design.

**Fig. 25:**
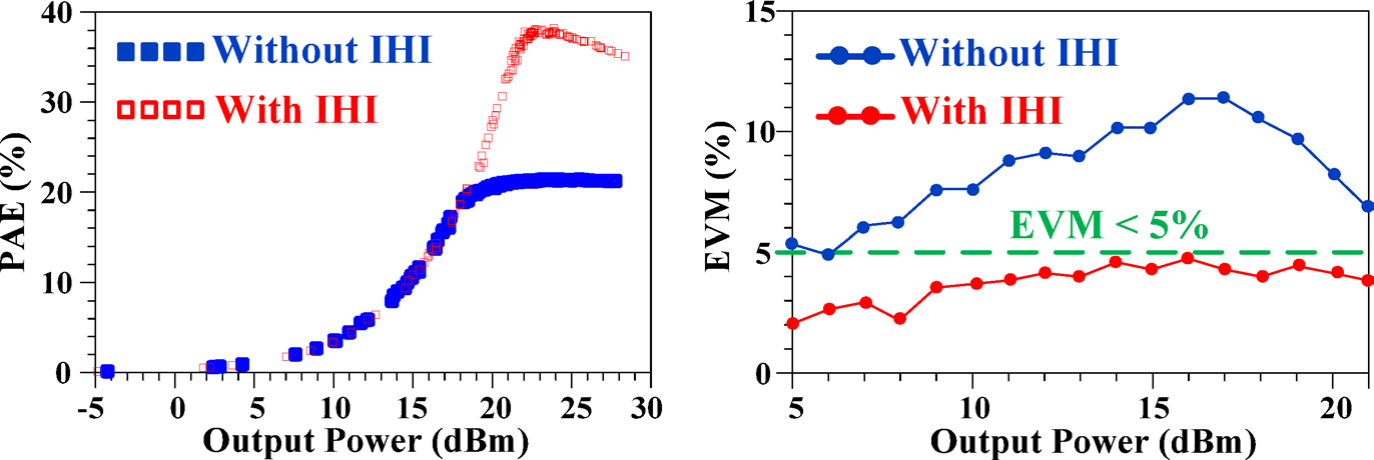
VPSV-PA performance with and without proposed IHI method.

Figure 25 demonstrates the performance comparison of the VPSV-PA with and without the proposed IHI method under a modulated input signal at 1900 MHz. The simulation results clearly illustrate that implementing the VPSV significantly enhances the PAE, particularly at higher output power levels. Notably, while systems without VPSV experience a sharp decline in PAE at elevated power outputs, the use of VPSV effectively mitigates this drop, maintaining high efficiency across a broader range of output powers. However, while VPSV improves efficiency, it shows limitations in addressing the Error Vector Magnitude (EVM), a critical factor for signal integrity. The integration of the IHI technique proves instrumental here, as it simultaneously improves both EVM and efficiency. Remarkably, the EVM consistently remains below 5%, a significant improvement compared to systems without IHI. This dual enhancement in both efficiency and signal quality underscores the performance of the proposed structure, demonstrating its potential for advanced applications in PA designs.

**Fig. 26:**
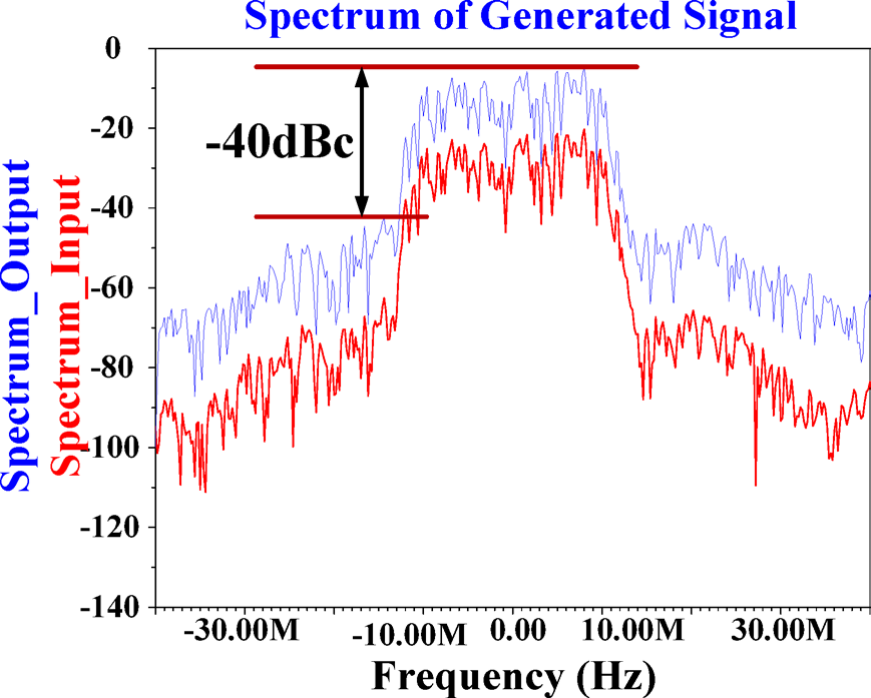
Adjacent Channel Power Ratio (ACPR).

**Fig. 27:**
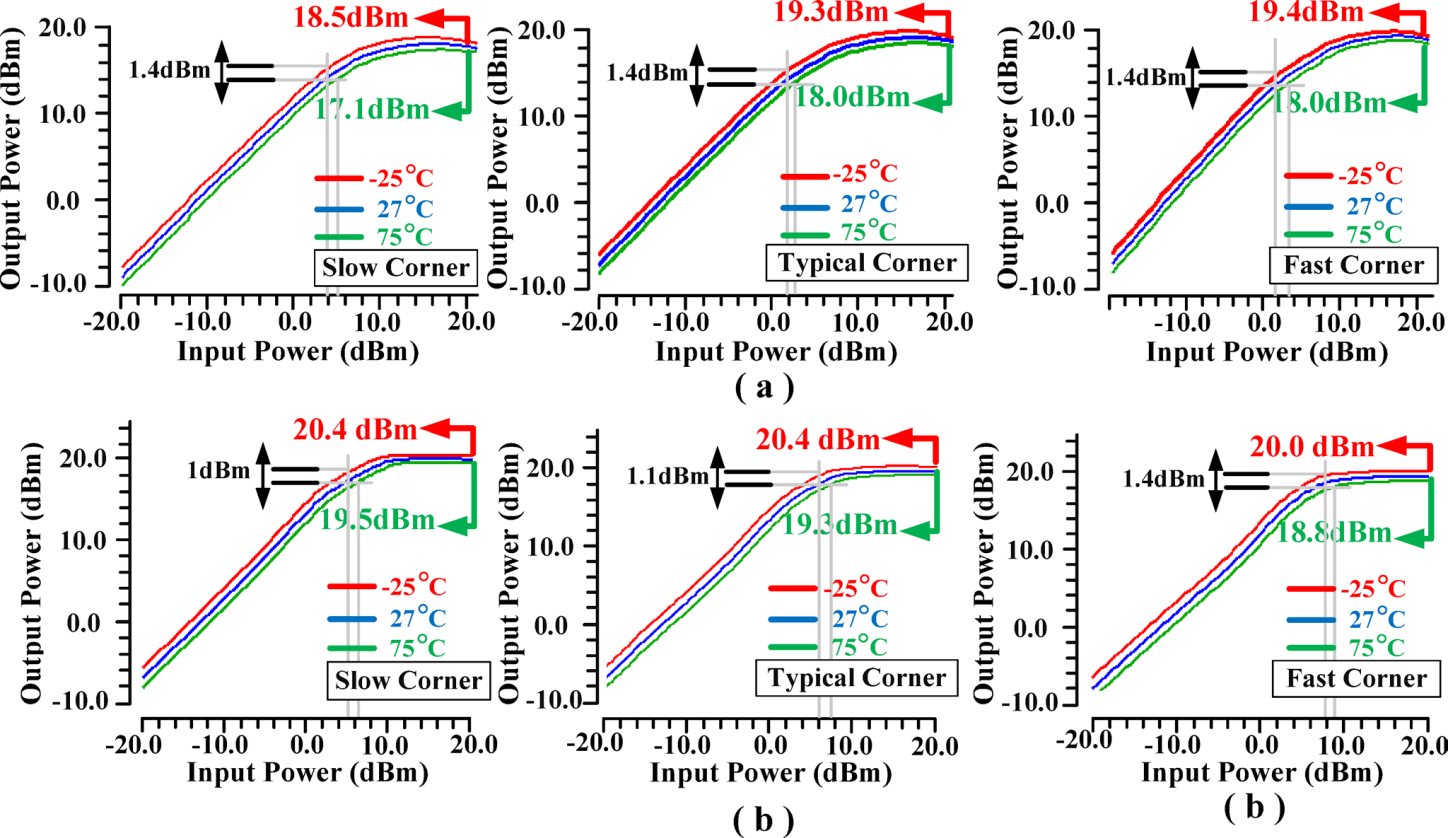
Effects of temperature and different corners of the output power: (**a**) without (**b**) with the IHI method.

Figure 26 illustrates the Adjacent Channel Power Ratio (ACPR) performance of the proposed PA, showing a value of approximately − 40 dBc. Figure 27 illustrates the impact of varying temperature conditions and corner variations on the output power performance of the proposed PA. The figure showcases the most challenging scenarios both corner and temperature fluctuations across a broad operational range. While some of these extreme conditions may not be encountered in practical applications, they have been deliberately considered to rigorously validate the robustness and accuracy of the proposed methodology. In the PA design without the proposed IHI method, the P_1dB_ under optimal conditions is approximately 15.1dBm. However, by employing the suggested IHI technique, even in the worst-case scenario, the P_1dB_ improves significantly to approximately 17.3dBm, demonstrating a notable performance enhancement. This result highlights the effectiveness of the proposed structure, as the P_1dB_ and maximum output power are consistently higher compared to the conventional design, even under the most extreme corner and temperature conditions. The proposed IHI approach not only ensures the P_1dB_ values across all operational scenarios but also guarantees a substantial increase in maximum output power, demonstrating its robustness and reliability. These improvements underscore the potential of the IHI technique as a solution for the PA operation, offering both enhanced performance and resilience under challenging operating conditions.

**Fig. 28:**
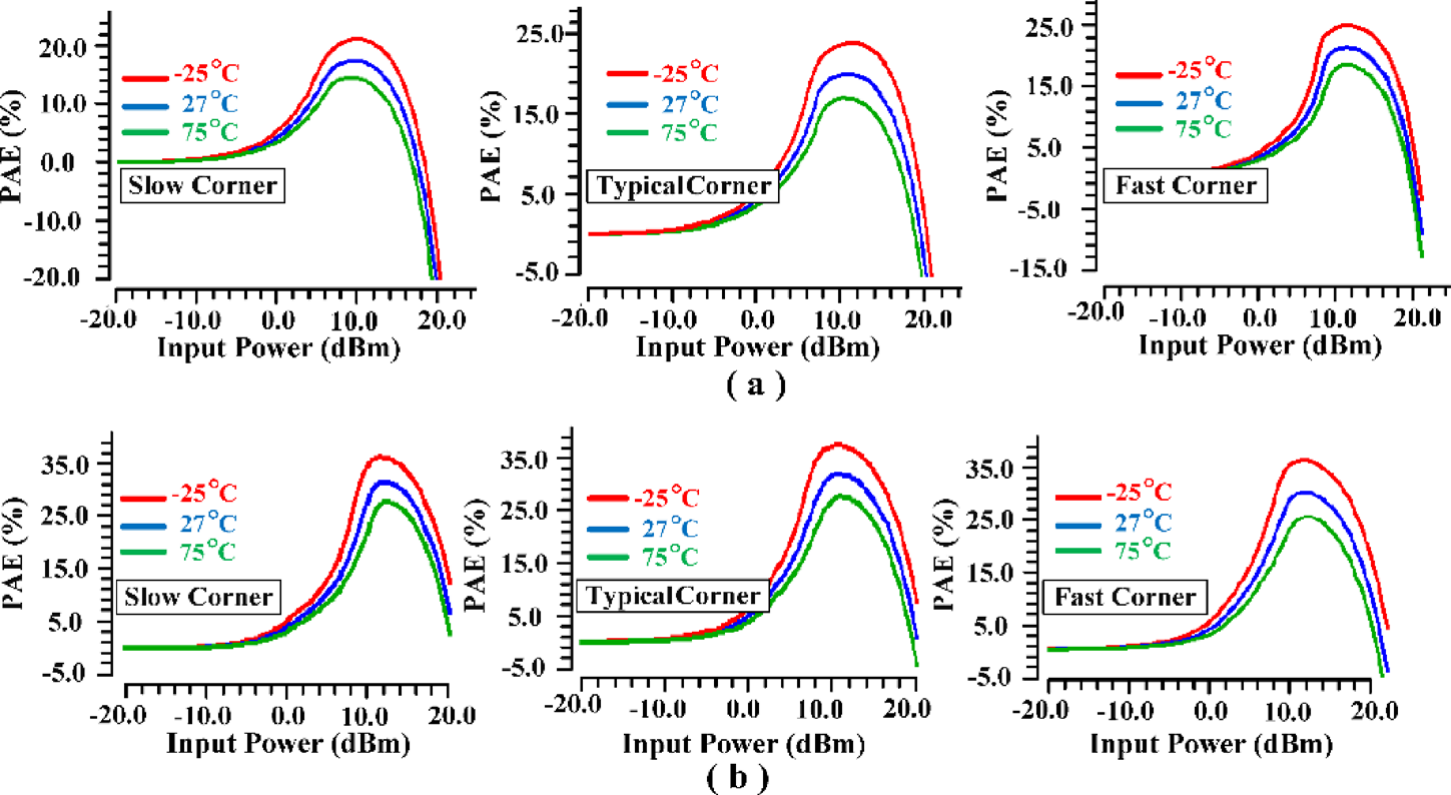
Effects of temperature and corner variations on Power Added Efficiency (PAE): (**a**) before (**b**) after employing the suggested IHI method.

It is crucial to analyze the influence of temperature and process variations on the PAE, as demonstrated in Fig. 28. The simulation results reveal a significant enhancement in efficiency at the P_1dB_ following the implementation of the IHI method. Notably, Fig. 28 illustrates a marked reduction in PAE variation with this approach, indicating improved stability and reliability in performance. These findings underscore the robustness of the proposed method, showcasing its effectiveness in optimizing PA performance under varying operational conditions.

**Fig. 29:**
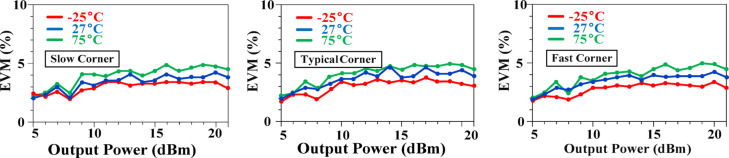
Effects of temperature and corner variations on EVM.

Figure 29 demonstrates the impact of varying temperatures and corners on the EVM performance of the proposed PA. Even under the most challenging conditions, including extreme corner variations and temperature fluctuations, the EVM remains below 5%. This consistent performance across a wide range of scenarios highlights the robustness of the proposed technique, underscoring its potential to deliver high-quality signal integrity and reliable PA performance.

**Fig. 30:**
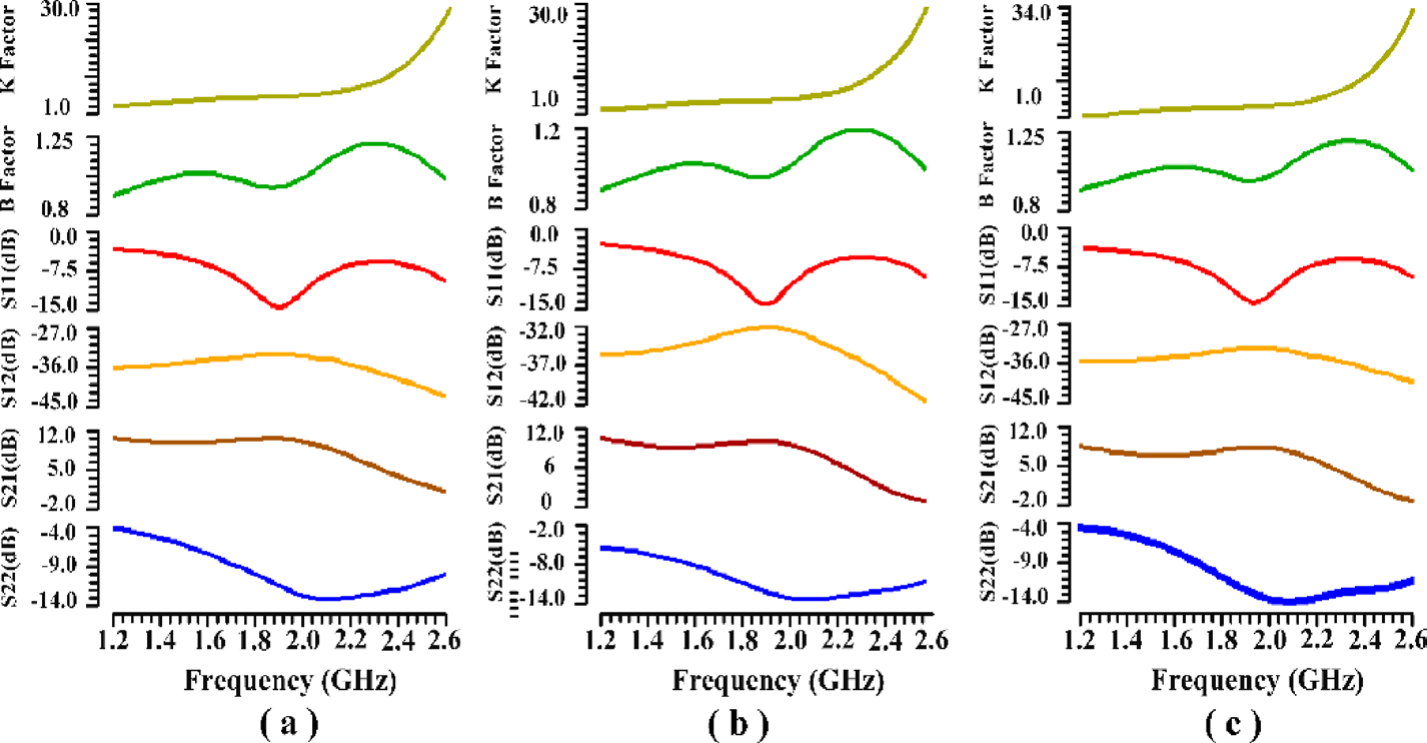
Scattering parameters performance (Stability factors, S11, S12, S21, S22) in different power levels around P_1dB_: (**a**) Pin: 5dBm (**b**) Pin: 6.9dBm (**c**) Pin: 8dBm.

Figure 30 illustrates the scattering parameters, including the stability factors (β and κ), S11, S12, S21, and S22, for the proposed PA at different input and output power levels. Even under the most unfavorable conditions, S11 and S22 remain below − 10dB within the frequency range of 1.8 GHz to 2 GHz. The stability factor for the proposed PA using the suggested IHI technique is evaluated based on the stability equation from reference^45^, which defines the stability factors as follows:

28 $$\:Stability\:Factor\:\left({\upbeta\:}\right)=1+{\left(\left|{S}_{11}\right|\right)}^{2}-{\left(\left|{S}_{22}\right|\right)}^{2}-{\left(\left|{S}_{11}{S}_{22}-{S}_{12}{S}_{21}\right|\right)}^{2}$$

29 $$\:Stability\:Factor\:\left({{\rm\:K}}\right)=\:\:\frac{1-{\left(\left|{S}_{11}\right|\right)}^{2}-{\left(\left|{S}_{22}\right|\right)}^{2}+{\left(\left|{S}_{11}{S}_{22}-{S}_{12}{S}_{21}\right|\right)}^{2}}{2\left|{S}_{12}{S}_{21}\right|}$$

The PA remains unconditionally stable when both the β-factor and κ-factor are greater than zero and one, respectively^45,46^. As shown in Fig. 30, the proposed PA, utilizing the IHI technique, exhibits unconditional stability across the frequency range of 1.8 GHz to 2 GHz. The scattering parameters around P_1dB_ have been investigated to examine the effects of the proposed method, confirming that the PA remains unconditionally stable with the proposed technique.

**Fig. 31:**
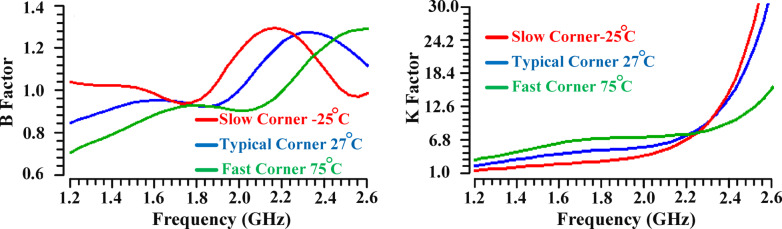
Effects of temperature and process variations on stability factor with the proposed method.

Figure 31 illustrates the impact of varying temperatures and corner conditions on the stability of the proposed PA. The simulation results confirm that the proposed PA maintains unconditional stability across all examined conditions, highlighting its consistent performance in diverse operating scenarios.

**Fig. 32:**
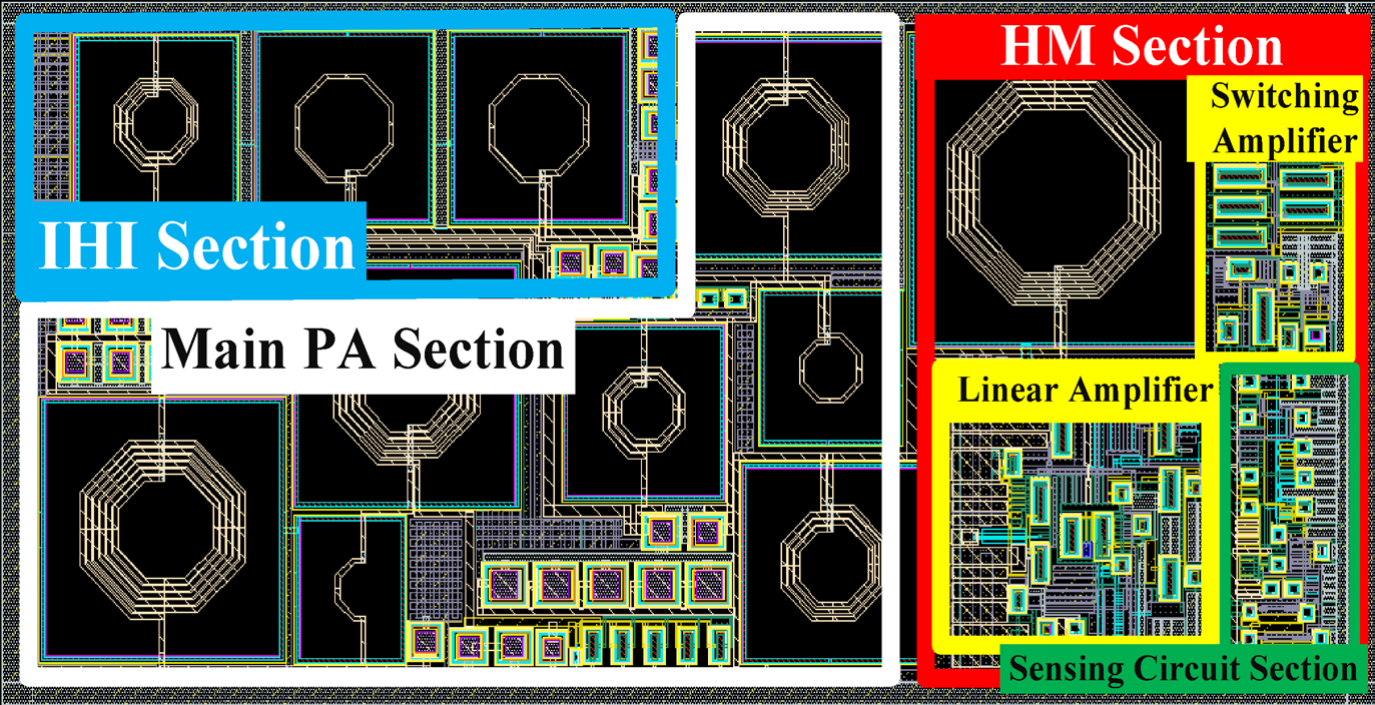
Proposed fully integrated IHI PA along with the HM section.

Figure 32 presents the proposed fully integrated IHI PA along with the HM section, occupying an area of approximately 1.67 mm×0.8 mm (1.34 mm²) on the chip. The IHI and HM sections account for approximately 27% and 34% of the total area, respectively, and are fully compatible with the CMOS process.

Table 2 presents a comprehensive comparison of the proposed PA with similar designs. To ensure a fair comparison, the table focuses on applications with comparable constraints and performance requirements. The proposed PA, utilizing the IHI technique, shows significant improvements across key performance metrics such as P_sat_, P_1dB_, PAE, and EVM. Specifically, the IHI method results in a notable increase of 0.6dB in gain, a 1.2dBm improvement in P_sat_, and an outstanding 4.4dBm boost in P_1dB_. Furthermore, the proposed technique achieves an approximately 12.5% improvement in maximum PAE, and a remarkable 15% increase in PAE at P_1dB_. Additionally, the technique successfully reduces the EVM from a range of 5–10% to below 5%, further underscoring its effectiveness in enhancing the PA’s overall performance and signal integrity. One of the key distinctions of this work compared to others is its ability to achieve these improvements with a supply voltage of only 1.8 V. Many similar designs, as shown in the references, operate at supply voltages exceeding 3 V to reach comparable output power levels. For instance, references^11,45^, and^48^ utilize higher voltages or BiCMOS technology to achieve their results. In contrast, the proposed PA achieves high performance with a lower supply voltage, making it not only more energy-efficient but also highly suitable for integration in CMOS processes. Furthermore, while conventional architectures often rely on external elements and techniques such as digital predistortion (DPD), transformers, and power combiners to enhance linearity and output power, the proposed design eliminates the need for these components. As a result, the IHI-based design maintains a fully integrated architecture, significantly reducing chip area and complexity. The absence of off-chip elements and the use of a rail-to-rail architecture with constant G_m_ in the input stage further underscore the integration potential of the proposed solution. This stands in stark contrast to designs such as^45^, which employ off-chip DPD, and^47^, which uses resistor-based structures that complicate the architecture. Additionally, the proposed HM design avoids the drawbacks associated with conventional HM approaches that utilize resistor-based structures or digital controlling circuits, such as ADCs and DACs. The proposed solution offers a much simpler and more efficient alternative, making it ideal for modern applications that demand high integration and performance. Based on the Figure-of-Merit (FOM) metrics outlined in references^50^, the proposed PA achieves the highest FOM among the compared designs. This not only highlights the robustness and efficiency of the IHI method but also positions the proposed PA as a highly competitive and effective solution for advanced wireless communication systems.

**Table 2:** Performance comparison between proposed PA and previous Works.

Reference Specifications	This work			^45^	^47^*	^46^	^11^	^48^	^49^
	Without IHI	With IHI	Improvement						
CMOS Tech. (nm)	180	180	----	BiCMOS	180	350	pHEMT	65	180
Frequency (GHz)	1.9	1.9	----	2.4	1.85	1.9	1.95	1.8	1.8
Supply Voltage (V)	1.8	1.8	----	3.3	3.3	4.2	3.4	3.3	3.3
Gain (dB)	13.2	13.8	+ 0.6	11	7	16.5	14	12.5†	12
P_1dB_ (dBm)	14.3	18.7	+ 4.4	18†	20	12†	12.5†	20†	21†
P_sat_ (dBm)	18.6	19.8	+ 1.2	20	22	18	16	21.3	23.5
PAE @ P_1dB_ (%)	7.3	21.9	+ 14.6	15†	22†	7†	15†	16	16†
Max. of PAE (%)	19.9	32.4	+ 12.5	28	26	22	24	18	21.6
Area (mm^2^)	$\:\sim$1	1.34	----	4	0.87	1.65	9	5.2	1.242
EVM (%)	5–10	< 5	~ 5	5	N/A	1.1	12.5†	8	$\:\sim$6
Architecture	Standalone PA + on-chip Matching	ET IHI PA based-on sensing circuit + on-chip Matching + without off-chip elements (Fully Integrated)	----	ET PA based-on sensing resistor with off-chip elements	ET common drain differential PA based-on switching modulator with transformer matching	ET cascode differential PA based-on sensing resistor with two off-chip baluns	Multiple-stage PA with directional coupler and variable power supply	ET cascade differential PA with four transformers and third harmonic trapping	Differential cascade PA with input and output transformers and RC feedback
^(1)^ FOM_sat_	50.4	54.3	✓	53.1	48.5	53.5	49.6	51.5	53.9
^(2)^ FOM_1dB_	46.1	51.5	✓	48.4	45.8	42.5	44.1	49.6	50.1

† Graphically estimated * Simulation Results.

$$\:\left(1\right)\:{FOM}_{\:\:sat\:}\:\:=\:{P}_{sat}\:\left[dBm\right]\:\:+\:Gain\:\left[dB\right]\:+\:20\:Log\left({f}_{C}\left[GHz\right]\right)\:+\:10\:Log\left({PAE}_{max}\right[\%\left]\right)\:\:\:\:$$^50^.

$$\:\left(2\right)\:{FOM}_{\:\:1dB\:}\:=\:{P}_{1dB}\:\left[dBm\right]\:\:+\:Gain\:\left[dB\right]\:+\:20\:Log\left({f}_{C}\left[GHz\right]\right)\:+\:10\:Log\left({PAE}_{1dB}\right[\%\left]\right)\:\:\:\:$$.

## Conclusion

This paper presents a highly efficient and linear fully integrated PA with significant performance improvements, achieved through the introduction of a novel IHI technique. Unlike conventional RF systems that treat harmonics as detrimental nonlinear factors and typically employ filters to remove them, the proposed method embraces internal harmonics to enhance PA performance. By leveraging this unconventional approach, this work proposes a new analytical framework for analyzing the impact of internal harmonics on PA behavior and introduces a mathematical model that deepens the understanding of harmonic effects. The proposed IHI method not only eliminates the need for complex components like D/A or A/D converters, transformers, circulator, power combiners, and splitters, which are commonly used in conventional linearization techniques, but also simplifies the architecture by removing the dependency on external auxiliary RF sources. Furthermore, this paper introduces a new HM architecture to improve PA efficiency by implementing a VPSV rather than a fixed power supply. The proposed architecture is based on a comprehensive analysis of the relationship between the switching frequency of the HM and the inductor value, leading to an efficient solution for modern PA designs. The proposed design also stands out by integrating a fully on-chip inductor, offering a more compact and efficient solution than conventional designs that rely on external components. The HM uses an ultra-wideband LA with constant G_m_, which significantly reduces distortions and enhances signal tracking accuracy for high PAPRs and wide bandwidths. Moreover, the novel SCS integrated into the HM provides the necessary voltage for the SA based on current, without the need for external reference voltages. Compared to standalone PAs, the proposed design demonstrates impressive improvements in key performance metrics, including a 1.2dBm increase in P_sat_, a 4.4dBm improvement in the P_1dB_, a 12.5% increase in maximum PAE, and a 14.6% improvement in PAE at P_1dB_. Additionally, the EVM is reduced to below 5%, confirming the linearity and efficiency of the proposed PA. In conclusion, the integration of the IHI technique and the new HM design makes this fully integrated PA a highly competitive solution for advanced wireless communication systems. The proposed design not only achieves high performance metrics but also offers a simplified, highly integrated architecture, making it well suited for modern communication standards that require high efficiency, linearity, and integration. One of the limitations and challenges of the proposed architecture is the use of a relatively large number of inductors in the matching networks and as RF chokes. This leads to two main issues: first, a considerable area is occupied on-chip; second, due to the limited quality factor (Q) of on-chip inductors, power losses increase, which can reduce overall efficiency. While using off-chip inductors can reduce losses and improve efficiency, this would come at the expense of losing a fully integrated design. Alternatively, some on-chip inductors could be partially replaced by transmission lines, which act as distributed reactive elements at high frequencies. Transmission lines can offer higher quality factor (Q) compared to on-chip inductors and may reduce power losses. However, their effectiveness strongly depends on the operating frequency: at lower frequencies, the required line length for equivalent inductance becomes large, which can significantly increase the occupied chip area. Additionally, transmission lines cannot provide ideal RF choke behavior for DC biasing, and integrating them within a fully on-chip design may be challenging. Therefore, while transmission lines provide a potential option for loss reduction at high frequencies, they introduce trade-offs in terms of chip area, DC bias compatibility, and overall integration. In summary, the choice between on-chip inductors and transmission lines involves a balance between integration, efficiency, and layout constraints, and represents a fundamental trade-off in the proposed architecture. Looking ahead, future work will focus on fabricating the proposed architecture in a 180 nm CMOS process to validate the results under real-world conditions. This includes testing with 5G test signal regimes, such as OFDM signals with high PAPR (e.g., 11–13 dB) and bandwidths up to 400 MHz, as well as carrier aggregation scenarios. These efforts will further demonstrate the robustness of the IHI and HETM in practical 5G/6G applications, addressing the current limitation of simulation-only validation.

## Data Availability

The data used to support the findings of this study are available from the corresponding author upon request.
